# Provenance of late Pleistocene loess in central and eastern Europe: isotopic evidence for dominant local sediment sources

**DOI:** 10.1038/s41598-024-83698-5

**Published:** 2025-01-10

**Authors:** K. Fenn, I. L. Millar, A. Bird, D. Veres, Doris Wagner

**Affiliations:** 1 Department of Geography and Planning, University of Liverpool, Liverpool, L69 7ZT UK; 2 Geochronology and Tracers Facility, British Geological Survey, Keyworth, Nottingham, NG12 5GG UK; 3 School of Environment Sciences, University of Hull, Hull, HU6 7RX UK; 4 Institute of Speleology, Romanian Academy, Clinicilor 5, 400006 Cluj-Napoca, Romania

**Keywords:** Geochemistry, Palaeoclimate, Geochemistry, Geomorphology, Sedimentology

## Abstract

Loess profiles along the Danube River provide a record of long-term Quaternary dust (loess) deposition in central-eastern Europe. Here, Sr–Nd isotopic data from four loess-palaeosol profiles (47 samples) spanning the last two-glacial-interglacial cycles are presented. The isotopic compositions generated by this study are compared with bedrock and sedimentary samples from Europe and North Africa to decipher the sources of sediment. The results demonstrate that over the last 300 ka the alluvial plains of the Danube (which are themselves sourced from surrounding mountain belts) are a local source of material and consequently sediment experiences aeolian transport over relatively short distances. The results dispute the commonly held assumption that the Sahara was a sediment contributor to loess in central-eastern Europe as North African contributions are not needed to explain loess signatures. Consequently, the findings suggest a suppressed southerly wind direction and dominance of the westerly and north-westerly wind systems over the entirety of the record.

## Introduction

Atmospheric mineral dust is not only an integral part of atmospheric systems dynamics^1^, but also a key element of the biogeochemical cycles^2^, as well as more broadly the biosphere, hydrosphere and cryosphere^3,4^. It has also been shown to be vital during global climate reorganisations, such as glacial terminations^5^. Despite their importance, modern dust distribution and fluxes are poorly understood due to quantification challenges, limited observational capabilities, and spatial and temporal heterogeneity^6–8^. These challenges can be addressed through studies of loess deposits; continental, geological archives of aeolian mineral dust^9^. In particular the identification of loess source areas and through them dust transport pathways remains critical for understanding atmospheric circulation patterns, the nature of transported dust, deposition modes, and interaction between dust cycle and climate^10,11^.

**Fig. 1:**
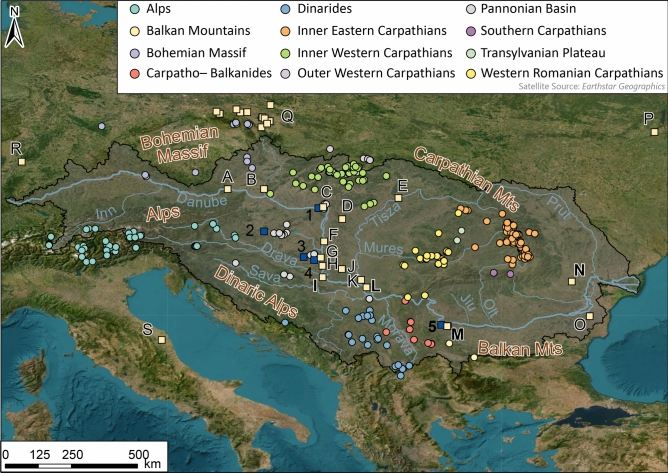
Location of fluvial sands and silts (blue squares), loess (yellow squares), and bedrock (coloured circles) samples. New samples generated in this study are marked in bold. Additionally, loess profiles mentioned in the text that fall within the catchments of the Danube and its main tributaries mentioned. Capital letters refer to loess profiles A—Krems, B—Grub, C—Basaharc, D—Mende, E—Tokaj, F—Paks, G—Dunaszekcső, H – Zmajevać, I—Erdut, J—Crvenka, K—Titel, L—Surduk 2, M—Slivata 1 and 2, N—Balta Alba Kurgan, O—Urluia, P—near Kiev, Q—collection of Polish sites, R—Nussloch, S—Ponte Crispiero. Numbers refer to river sites: 1—Basaharc fluvial sands, 2—Baltavár fluvial sands, 3—Puszta fluvial sands, 4—Mohács fluvial sands, 5—Danube. For source metadata see Supplementary Table 1. Map generated using ArcGIS PRO.

Loess deposits in central and eastern Europe within the Danube basins (Fig. 1) span (semi-) continuously for at least 0.7 Ma^12,13^ and are therefore an important climate archive of Pleistocene and Holocene and changes in atmospheric circulation during this time. Provenance through means of geochemistry of Danubian and Pannonian Basin loess been a research focus for over a decade^14–17^, as reconstructing sediment sources has implications for interpretations of paleoenvironmental signals preserved within loess^9^ and modelling of dust transport and fluxes^11^. However, the lack of consensus over the sources of sediment for Danubian loess still persists, with one hypothesis based on grain size and satellite observations of modern dust storms suggesting that the Saharan Desert was a major contributor of fine grain material to Danubian loess^18,19^. This in turn impacts understanding of transport mechanisms and circulation patterns. Some argue that contributions are increased during interglacial periods^18,20^ and that Saharan dust input could contribute as much as 40% of the total fine grained material to the soils/palaeosols^19,21,22^. Moreover it has been suggested that during glacial periods dust storms coming over from North Africa were more frequent^23^ which would also explain larger contributions during glacial periods. This scenario implies dominant southerly winds (with potential shifts to the westerly winds positions and patterns) and much larger volumes of dust fluxes which would have to be considered in climate models. However there is growing evidence for an alternative hypothesis; the Danube (and its tributaries) alluvium is the most immediate geomorphic source of fine grained sediment for loess^9,17,24^. Grain size, U–Pb geochronology and Hf isotopes of detrital zircon, and bulk elemental chemistry supports this argument, and show that the surrounding mountain belts (the Alps, Bohemian Massif, Carpathians, Dinarides, and Balkan Mountains) were the primary sources of silt in Danube alluvium^15,16,25,26^. This scenario fits with modern observations of the core atmospheric patterns and paleo wind reconstructions of predominant westerly and north-westerly wind direction and southern displacement of westerlies as a consequence of ice-sheet growth^26–29^. It also supports the predominantly short distance transport of large volumes of dust^15,17,30–32^. However European mountain belts in the region have a broadly consistent origin, having predominantly formed from peri-Gondwanian terranes, and sediments accumulated at the continental margins of Gondwana^33–35^ which results in similar geochemical signatures. Consequently disentangling individual source contributions and their proportion to loess deposits remains unknown, impacting understanding of sediment generation mechanisms, and sediment transport pathways. Thus, the debate over the sources of sediment for loess in central and eastern Europe persists, and until settled the modes of dust transport and their impact on atmospheric processes remain poorly understood.

Studies of loess sources have used a variety of techniques, including major and trace element chemistry^17,36^, zircon U-Pb^14,25,37,38^, zircon Hf isotopes^15,25^, magnetic properties^39,40^, and radiogenic isotopes like Nd and Sr^14,41^. Single grain methods such as zircon U–Pb, when compared to bulk sediment methods (such as major and trace element analysis and radiogenic isotopes^42,43^) have less difficulty in averaging source inputs but can bias loess provenance interpretations based on source lithology zircon fertility^14,15,43^. Major and trace element analysis and radiogenic isotopes (e.g. Nd, Sr, Hf) have an advantage in their ability to distinguish source information from different grain-size fractions^44,45^. Radiogenic isotopes also provide a basis for developing mixing lines between potential sources, and consequently the proportions of source contributions can be identified. Here new Sr–Nd isotopic data for 47 loess samples (bulk sediment and < 2 µm) from four loess sites are presented and compared to published source data to examine loess formation, constrain dust transport pathways, and examine implications for dust and past climate links within the Danube loess field.

## Study area

The loess deposits of Central and Eastern Europe (Fig. 1) fall under seasonally competing influences of the Atlantic, Continental and Mediterranean air masses, the large scale dominance of which has dramatically changed over glacial to interglacial cycles^46^. The climatic influences are (and were) further modulated by the surrounding mountain belts (the Alps, Bohemian Massif, Carpathians, Dinarides, and Balkan Mountains). Climate reconstructions show cold climatic conditions during glacial periods^46–48^ though some smaller areas within the region acted as refugia^49^. Humidity reconstructions show a strong north–south gradient, with southern regions experiencing near arid conditions. This variable humidity in part played a role in the observed shifting dust fluxes across the region^50,51^.

The loess–palaeosol sequences investigated as a part of this study are located along the Middle and Lower reaches of the Danube River (Fig. 1) in Croatia (Erdut), Serbia (Surduk 2), Bulgaria (composite profile of Slivata 1 and 2), and Romania (Balta Alba Kurgan). The geographical spread of the sites captures a range of potential sources contributing sediment to the loess profiles. Most of the sections (apart from Balta Alba) are located in the vicinity of the main fluvial channel of the Danube and rest on fluvial terraces. The studied sequences capture a sedimentary history spanning the last two glacial cycles; Erdut 75–231 ka^52^, Surduk 2 19–52 ka^51^, Slivata 1 and 2 14–95 ka^50^, and Balta Alba Kurgan < 8 ka^53^. Details of individual samples used in this study (including ages and sedimentary units), are presented in Supplementary Table 2. Additionally to supplement the loess dataset and provide a comparison a sample was collected from the Danube’s modern alluvial sediment (Fig. 1 sampling point 5).

## Results

**Fig. 2:**
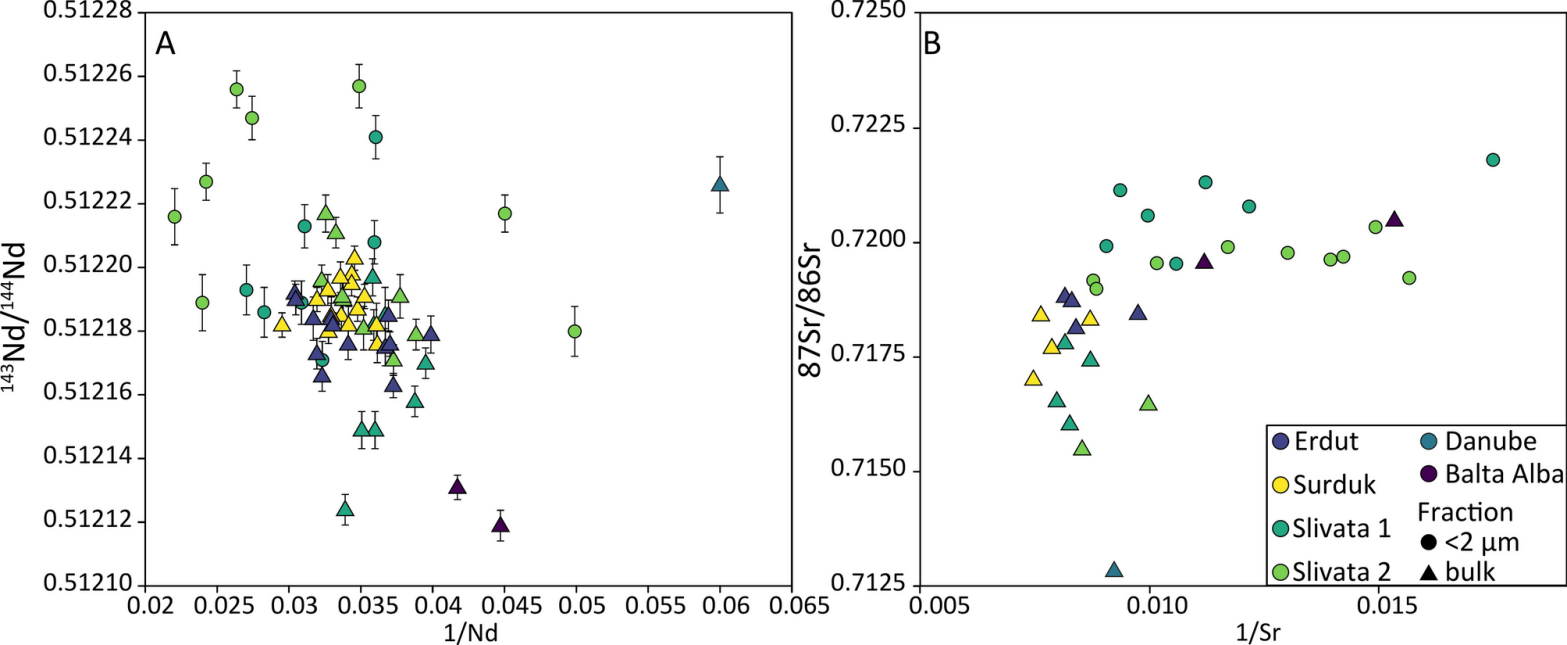
Isotopic ratios plotted against inverted concentrations for analysed loess and Danube modern alluvium samples. Error bars (2 standard deviations) are shown in both panels; however, Sr errors are negligible. **A** ^143^Nd/^144^Nd vs 1/Nd. **B** ^87^Sr/^86^Sr vs 1/Sr. Bulk sediment (triangles) and < 2 μm fraction (circles).

^143^Nd/^144^Nd ratios, from this study, show a spread of values from 0.5121 to 0.51226 (Supplementary Table 3 and Fig. 2A); with a range of 0.51212–0.51222 (εNd -8.2 to -10) for bulk sediment and 0.51211–0.51226 (εNd − 7.2 to −10.3) for < 2 μm fraction. By comparison the differences in Nd concentrations are relatively constrained and vary between 16 ppm (Danube) and 45 ppm (Slivata 2). Most bulk samples (Erdut, Surduk 2, and Slivata 2) cluster in the centre of the diagram. However Slivata 1 has a much greater spread in ^143^Nd/^144^Nd ratios and together with Balta Alba Kurgan have some of the least radiogenic values. Fine grained (< 2 μm) fractions have a much more varied range of values in the Nd concentrations relative to bulk sediment.

The ^87^Sr/^86^Sr values range from 0.71283 to 0.72468, with the lowest value measured for the modern Danube and highest for Balta Alba Kurgan (Supplementary Table 3). Figure 2B shows there are two more distinct clusters separating bulk and < 2 μm fraction, with ranges of 0.71283–0.72048 and 0.71877–0.72468 for bulk and < 2 μm respectively. Bulk sediment shows narrow Sr concentration values but a much more diverse range in the ^87^Sr/^86^Sr data. Conversely, the < 2 μm (clay) fraction shows limited variability in Sr isotopes but a large variability in Sr concentrations suggesting a link between grain size and strontium isotopic values. Moreover the spread of isotopic values in Fig. 2A and B suggests a degree of mixing between potential source regions.

## Discussion

**Fig. 3.**
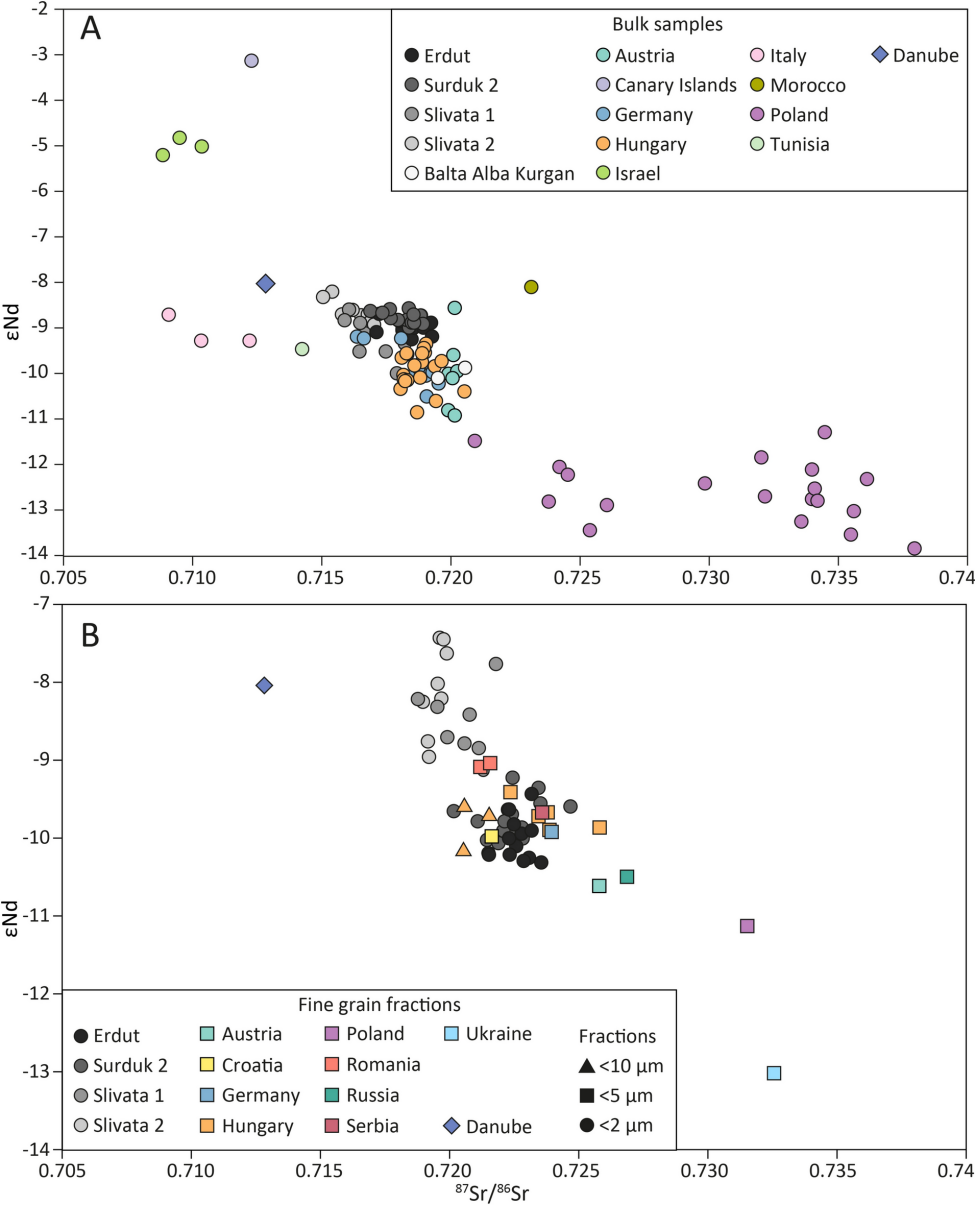
^87^Sr/^86^Sr vs εNd for European, Mediterranean, and North African loess **A** shows results for available bulk samples and **B** for a range of fractions. Additionally the Danube’s modern alluvial bulk material is plotted. Samples analysed in this study are plotted as individual sites, whilst published samples are colour coded by country for clarity (see Supplementary Table 1 for details).

Figure 3 shows bulk and < 2 µm samples compared with other European, Mediterranean, and North African loess isotopic signatures. A commonly observed ^87^Sr/^86^Sr offset between isotopic values of bulk samples and fine grain fractions is noted^14,41,54–56^; with bulk displaying broadly less radiogenic values. The difference between bulk and < 2 µm for sites analysed in this study ranges from 0.0033–0.0042. Strontium isotopic partitioning with grain size is well documented and relates to weathering and/or mineral composition though some argue that the direction of a ^87^Sr/^86^Sr shift can be sample and site specific, depending on the environment and composition of the material, e.g. a relatively feldspar rich sample in a humid climate will be more strongly affected by chemical weathering than a relatively quartz-rich sample in an arid climate^57–60^. Feng et al. (2009) demonstrated a variation of up to ~ 0.012 for different grain size fractions of the same sample, thus it is likely that the Sr isotope offset noted in this study is a result of differences in mineral composition and chemical weathering^61–63^.

Conversely grain size exudes little influence on the Nd isotopic composition in aeolian sediments, making it a more reliable source rock tracer^59,64,65^. A distinction between bulk and < 2 µm fractions is observed across all investigated sites; however, the |εNd| difference ranges from 0.6 to 1.1, falling within the previously reported margin of error (ε1–2)^58,59,64,66^. Whilst the effect is negligible it is interesting to note that Erdut and Surduk 2’s εNd clay fraction is less radiogenic than bulk, whereas the opposite is seen in Slivata 1 and Slivata 2. Fine grain fractions at both Slivata sites have a slightly higher Nd concentrations in comparison to the bulk sample (~ 33 ppm and ~ 28 ppm respectively). This is likely driven by changes in the minerology between fractions (e.g. changes in clay types and/or Nd-bearing minerals like monazite or allanite), however as concentration values are not available for the < 2 µm fraction for Surduk 2 and Erdut this mineralogy link will have to be pursued with further analysis.

Overall Fig. 3 shows that fine and bulk fractions of Erdut, Surduk 2, Slivata 1, Slivata 2, and Balta Alba Kurgan have homogenous isotopic signatures and are similar to other Danubian loess (Austria, Hungary, Serbia, Romania). The plot does demonstrate Danubian loess has very similar isotopic signatures regardless of the fraction used and therefore is likely to share the same sediment source(s). For example Hungarian, Croatian, Serbian, and German samples all overlap in the εNd space and show a greater range of ^87^Sr/^86^Sr values and the same can be observed between Romanian and Bulgarian samples. It also shows that when sources are significantly different distinctions can be made between loess across Central Europe; for example, inputs from less radiogenic Scandinavian granitic rocks produce strongly negative εNd in Poland and Norway^67^ (Fig. 3A). The same can be noted for the finer fractions (Fig. 3B) e.g. Ukrainian and Russian loess samples show the most negative εNd values which likely represent inputs from less radiogenic Precambrian rocks in the East European Craton.

**Fig. 4:**
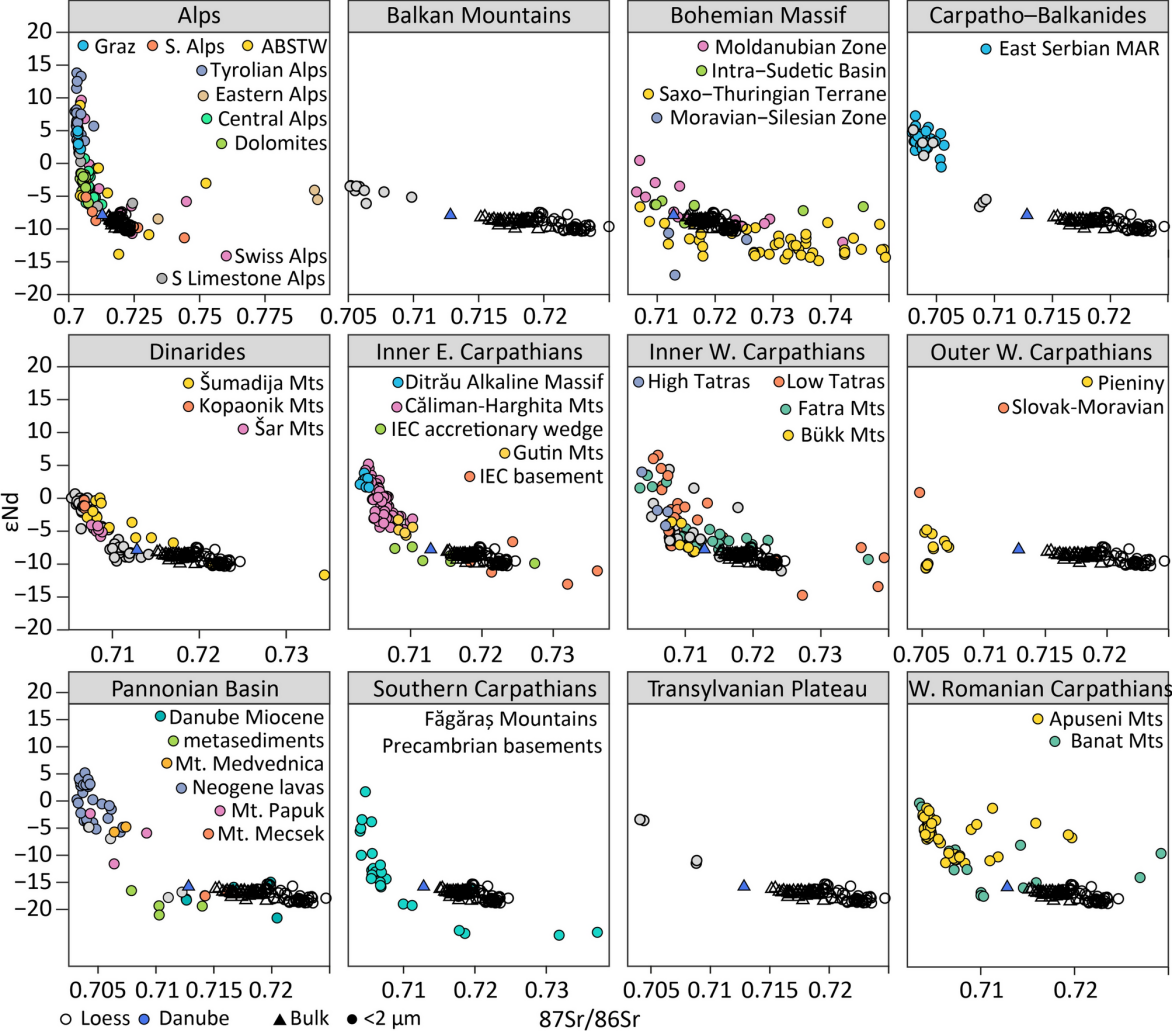
Sr–Nd isotope compositions of loess analysed in this study plotted with potential bedrock sources. Bulk sediment (triangles) and < 2 μm fraction (circles). Data are grouped by broad sources regions and colour coded by specific sources. Samples coloured grey represent a collection of samples that were taken across the region that cannot be grouped into a single location. Abbreviations: ABSTW—Austroalpine basement to the south of the Tauern Window (Eastern Alps), East Serbian MAR—East Serbian Mafic Alkaline Rocks, IEC—Inner Eastern Carpathians. Refer to Supplementary Table 1 for the details of source samples.

The εNd values of bulk material (Fig. 3A) for Erdut, Surduk 2, Slivata, Balta Alba overlap with published data for Austrian (Grub), German (Nussloch), and Hungarian (Tokaj) loess^44^ and seemingly suggest a common source for the sites. The overlap between Tokaj and Nussloch demonstrates geochemical similarity in prime source signatures (Carpathian for Tokaj and Alpine for Nussloch; Fig. 1) which in turn explains the similarity between these sites and Danubian loess. Further whilst these sites cluster (Fig. 3A) a trendline can be observed formed from Slivata 2 to Grub (Austria), suggesting a slight change in source material along the course of the Danube. For example, Slivata appears to receive greater contributions of more (less) εNd (^87^Sr/^86^Sr) radiogenic material from primitive sources such as East Serbian Cretaceous-Palaeocene Mafic Alkaline Rocks^68^ or Cretaceous Central Srednogorie volcanic rocks^69^ (Fig. 4). Contributions from these sources were previously identified by Fenn et al.^15^ through zircon U–Pb and Hf isotope analysis. These two approaches highlight the significance of smaller, often overlooked, contributions from regions such as Balkan Mountains or Dinarides in understanding provenance of Danubian loess. On the other hand Grub is located in proximity to the Bohemian Massif (Moravian Gap) and its less radiogenic Nd signatures are likely explained by the proximity to metamorphic rocks from Bohemia and older parts of Tatras (Fig. 1). Further downstream Romanian Balta Alba Kurgan shows less (more) εNd (^87^Sr/^86^Sr) radiogenic signatures indicating larger inputs of older more mature rocks such as the metamorphic basement of Eastern Carpathians (Fig. 4). However, as expected, this trendline between loess samples (Figs. 2 and 3) does not show a straightforward and clear downstream shift in loess isotopic values (i.e. Erdut–Surduk–Slivata 1–Slivata 2) as the bedrock changes are complex and often carry similar isotopic signatures. This also demonstrates that loess geochemical composition is not only related to changes in fluvial processes; changes in loess compositions are more irregular, e.g. Slivata 1 overlapping with Erdut. Nonetheless the results demonstrate that changes to contributions of minor bedrock sources occur and can be identified with a sufficient number of supplementary datasets, supporting previous work^17,70–73^. Whilst Fenn et al. (2022) suggested that minor spatial changes were possible these were hard to pinpoint due to similarities in zircon U–Pb ages across the region.

Figure 3 also shows a correspondence between the modern Danube’s fluvial samples and loess data, with fluvial samples offset along the strontium isotopic axis. This is likely explained by the grain size differences between alluvial plain sand dominated material (Supplementary Fig. 1) and silty loess. The Danube’s alluvium was collected ~ 10 km from the Slivata profile which explains its similarity to the Slivata loess in particular. Unfortunately fluvial Holocene and Pleistocene samples are not available for comparison with loess to make a definitive match between past Danube sediment and loess. However the fluvial transport mechanism (i.e. key role rivers play in delivering sediment from source regions) prior to loess deposition through aeolian redistribution of river sediments is well established theoretically^30,31,74,75^ and empirically^14,15,32,42^. Furthermore, the Danube Basin is a sink basin that begun forming with the closure of the Balkan fragment of Neotethys and Sava Ocean^76^ and entered a final evolution phase during the last 15–10 Ma. Over that time it has served as a relatively constant sedimentary sink to the surroundinguplifted mountain belts (i.e. Alps, Carpathians, Balkan Mountains)^77^. Therefore the material eroded, recycled, and transported by the Danube (and its tributaries) has relatively similar signatures, which was demonstrated by zircon U–Pb analysis of fluvial sediments across the region^78,79^ and similarity with loess signatures^14,15^. The overall similarity of Sr–Nd isotopes between loess sites (e.g. Austria, Hungary, Serbia, Bulgaria, Romania) and the modern Danube sediment further supports previous research which proposes the Danube’s sediment as the immediate source of fine grained sediment for loess profiles^14,15,17,25,31,80,81^.

To identify primary sources of loess material in Europe a range of datasets encompassing previously proposed potential bedrock source areas (the Alps, Balkan Mountains, Bohemian Massif, Carpathians, Dinarides, and Pannonian Basin) were assembled (Fig. 1 and Supplementary Table 1). Bulk and the < 2 μm fraction were plotted against 750 + published potential European bedrock source data points (Fig. 4). These provide strong constraints on Danubian loess samples and a wide range of potential sources.

Figure 4 shows that almost all potential source areas have heterogenous Sr and Nd isotopic values. These range from primitive volcanic rocks that have values similar to the mantle (strongly positive εNd), to rocks with evidence of continental crust melts assimilation, particularly evident in the Bohemian Massif and some Alpine samples. This variation is typical of mountain belts, which by their nature are made up of a range of materials accreted over time^35,82^. The loess compositions do not reflect this range in Nd and Sr values, suggesting that specific areas of given mountain belts are contributing sediment (based on past and modern drainage, erosion agents, variable erosion rates, bedrock resistance, etc.^83–87^) rather than whole mountain belts (e.g. Alps as a whole). Moreover while Figs. 2 and 3 showed there was fractionation between bulk and the < 2 μm fraction of loess, the groupings are not so obvious when compared to bedrock sources. Instead the bedrock, fluvial samples, and loess plot more as a gradient from bedrock to sedimentary deposits with the fluvial sample showing a less radiogenic Sr but a similar Nd isotopic signature to loess. This tendency was also reported by Fenn et al.^15^ where zircons data were analysed using Multi-Dimensional Scaling (MDS, Vermeesch 2013). The loess zircons from loess plotted in a cluster surrounded by samples from fluvial setting including, Danube, Drava, Tisza. Fluvial zircons in turn were surrounded by bedrock zircon data, indicating that they are more similar to the loess zircons than to those from the bedrock. The authors argued that these similarities result from multistage sediment transport from mass movements and glacial action at source, numerous fluvial transport cycles, and prior to the relatively short-distance, local aeolian transport. During these sediment transport cycles geochemical signatures are homogenised^15,70,88^ which consequently translates to the similarities between loess sites and progressive concentration of signatures from bedrock to loess. These conclusions are further supported by this study.

Only a few bedrock datasets in Fig. 4 show a direct overlap with loess, i.e. Făgăraş Mountains Precambrian basements (Southern Carpathians), Mecsek Mountains (Pannonian Basin), Palaeo-accretionary wedge and the metamorphic basement (Inner Eastern Carpathian), High Tatras and Fatra (Inner Western Carpathians), and some of the data from the Moldanubian Zone (Bohemian Massif, see Fig. 1). Whilst it is possible that these regions represent primary sources of sediment for loess, this is highly unlikely given their small areal extent across the basin and their location which would present some challenges in terms of sediment transport mechanisms and directions. For example, Făgăraş Mountains predominantly drain into the Olt River (Fig. 1) and Lower Danube and therefore cannot contribute material to any of the investigated sites. Mecsek Mountains in southern Hungary drain almost directly to the Danube. However, it is a very small mountain range with the highest point only ~ 680 m above sea level, which lacked conditions to not support glaciers. Thus the Mecsek Mountains were (and are) not capable of generating large volumes of sediment^89,90^. Both granitic rocks in Fatra Mountains have a very small overlap with loess (Fig. 4) suggesting they are just a minor contributor and unlikely a large dominant source. The metamorphic rocks in the Bohemian Massif (Moldanubian Zone; Fig. 4) might provide some samples that overlap well with loess; however it is unlikely that these represent large contributions of sediment to the Danubian systems given their limited spatial cover. These regions are therefore likely contributors of sediment that reinforces the "average Danubian signature”^15^.

**Fig. 5:**
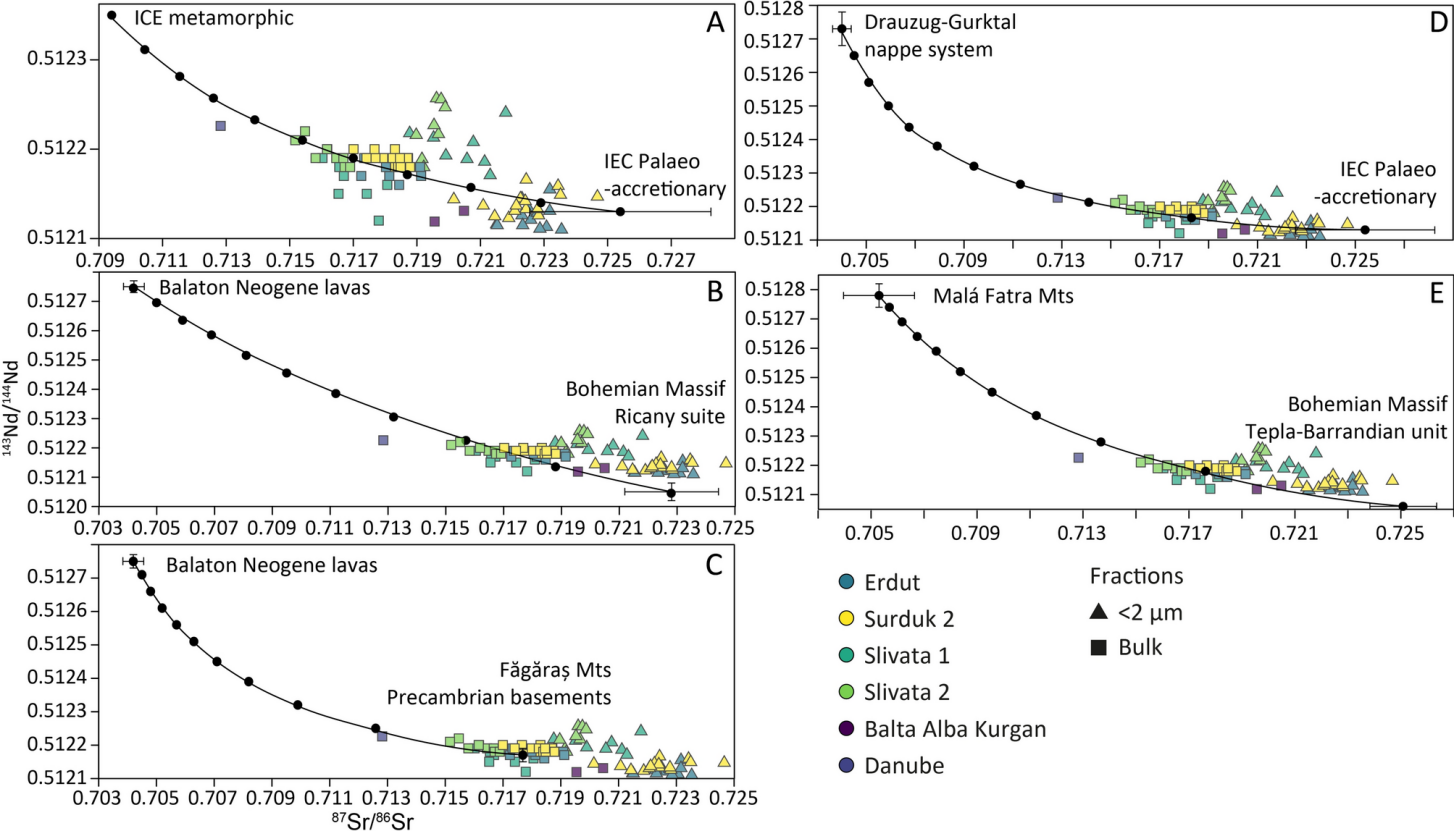
Mixing lines between averaged selected potential bedrock sources **A** Inner Eastern Carpathian paleo-accretional unit (flysch) and Inner Eastern Carpathian metamorphic rocks^91^; **B** Inner Eastern Carpathian paleo-accretional unit (flysch)^91^ and Drauzug-Gurktal nappe system^94^ in Alps; **C** Neogene volcanic rocks from Balaton^95^ and Ricany Suite in Bohemian Massif^96^; **D** East Serbian Mafic rocks^68^ and Inner Eastern Carpathian metamorphic rocks^91^; **E** Fatra Mountains in Western Carpathians^97^ and Tepla-Barrandian unit in Bohemian Massif^96^. In all cases error bars show standard deviation of each dataset. Mixing lines calculated following Faure (2001). For a full summary of explored end member combinations see Supplementary Figure 2.

Particularly interesting are the samples from the Eastern Carpathians (Bretila, Negrişoara, Rebra), representing metamorphic rocks and sedimentary rocks (flysch; palaeo-accretionary wedge)^91^. Both are located within the drainage of Mureş and Olt (Fig. 1) and represent pre-Gondwanian rocks of Cambrian to Ordovician in age^92^. Fenn et al. (2022) found large populations of 450 Ma zircons within Danubian loess, particularly in Serbian and Bulgarian samples^15^, however they could not attribute them to any source due to the lack of zircon U–Pb and Hf data from the Eastern Carpathians. Given the close isotopic match between loess and flysch, these rocks could represent the missing source of 450 Ma zircons. Sadly flysch, other sandy and fine-grained sedimentary rocks associated with marine deposition, and molasse are underrepresented in the isotopic, zircon, and elemental data. Yet they outcrop across Alps, Outer Carpathians, and the Pannonian Basin as a range of flysch units^93^. These rocks likely preserve a collection of geochemical signatures that correspond to millions of years of sedimentary erosion, transport and deposition; from the peri-Gondwanian margins to the closure of Tethys^76,93^. They are expected to have signatures associated with a wide range orogenic provinces, as a palaeo-accretionary wedge will include a mix of many different units (e.g. metamorphic and sedimentary lithologies, ranging from Cambrian to Ordovician). The fine-grained nature and abundance of flysch across Europe would make it an excellent source candidate for loess (Fig. 4), however there is a lack of available data to make a definitive association at this time. Notwithstanding the similarities between loess and some bedrock signatures discussed above, Fig. 4 shows that loess typically plots in between bedrock sources. Thus, the Sr and Nd isotopic signatures shown by loess are best explained by mixing between two (or more) sources; where one end member comes either from material dominantly sourced from radiogenic basic rocks, or directly from this type of material (primitive; positive εNd values), and a second end member that represents a “crustal” melt (negative εNd values). These trends were also observed in loess zircon Hf isotopic values reported by Újvári and Klötzli (2015) and Fenn et al. (2022). Mixing between multiple sources is not only supported by geochemical analysis but also sedimentary recycling and the multi-step delivery process that is closely related to the Danube and its tributaries^15,31^. Mixing averages were calculated for potential sources and plotted against each other to identify sources that explain loess isotopic signatures well. Figure 5 shows some of the most representative end-member combinations shown in Fig. 4. Of the published datasets investigated only a few had “crustal” (less radiogenic Nd and more radiogenic Sr) signatures required to explain the loess and Danube data, including the metamorphic basement and palaeo-accretionary wedge in Eastern Carpathians (Inner Eastern Carpathian), parts of Bohemian Massif, and Saxo-Thuringian Terrane (which are currently not within the Danube’s drainage). Conversely the primitive mantle melts signatures are abundant in almost all mountain belts surrounding the Danube River.

Figure 5A shows a mix between metamorphic rocks and sedimentary rocks (palaeo-accretionary wedge) from the Inner Eastern Carpathians^91^. This combination of isotopic signatures provides one of the best fits between loess and bedrock, with the mixing line running through the middle of bulk samples. In this scenario metamorphic rocks would contribute on average 30–40% but as much as 50% to some loess samples (Slivata 2, Bulgaria). However they do not explain the samples from Romania very well. Figure 5B shows a quite good fit between Inner Eastern Carpathians flysch^91^ and more radiogenic isotopic compositions. This fit is very representative of many “young” rock signatures seen across the basin, including East Serbian Mafic Alkaline Rocks, Neogene lavas, Inner Eastern Carpathians (Căliman-Harghita Mountains, Ditrău Alkaline Massif, Gutâi Mountains), Dolomites, Balkans, Tyrolian Alps, and Central Alps (Supplementary Figures). In all these cases these volcanic rocks explain at most 10–20% of contributions while flysch (or an as yet unknown contributor of similar signature) contributes between 80–90%. Figure 5C and E show mixing between rocks with young melt signatures, Neogene lavas from lake Balaton and East Serbian Mafic Alkaline Rocks^68^ respectively. Figure 5C shows a mix with Bohemian Massif rocks (Ricany suite), formed ~ 330 Ma as a part of the Central Bohemian Pluton^96^. One of the three dominant zircon populations found in Danubian loess is centred around 330 Ma^14,15,25^, and Fenn et al. (2022) proposing Bohemia as a potential source that could explain the Hf isotopic signatures within zircons. The results of this study support that hypothesis demonstrating that Bohemian rocks could account for as much as 80–90% of the Sr and Nd isotopic signatures in bulk samples. Figure 5D shows a mixing line with metamorphic rocks in the Eastern Carpathians, demonstrating a reasonably good fit for less radiogenic Nd signatures in loess. These young (60–70 Ma) volcanic rocks^68^ are spread out across the Carpatho-Balkanides and the drainage of the Lower Danube. Their isotopic signatures and geographic spread explains similarities with Bulgarian and Romanian loess samples, as previously noted by Fenn et al. (2020). Additionally the Precambrian basement located in the Făgăraş Mountains^98^ plots within loess Sr–Nd isotopic range and mixes well with almost any potential end member. Here they are shown with Neogene Balaton volcanic rocks. Finally the Fig. 5E panel shows another Central Bohemian Pluton^96^ unit (Bohemian Massif) mixed with Fatra Mountains (Western Carpathians) while fit is different, cutting in between Bulgarian/Romanian and Croatian/Serbian samples it still demonstrates a possible end-member combination. Crucially all panels of Fig. 5 show that large contributions are needed from the rocks which have less radiogenic Nd and more radiogenic Sr values (“crustal”) to explain the isotopic composition of loess. These typically explain between 50 to 90% of the loess signatures. Critically a range of end-members could be employed to generate mixing lines that fit well with loess Sr–Nd signatures. While it is possible that one of these mixes identifies two dominant primary sources of loess, it is almost impossible to pinpoint one. Given the range of signatures it is far more likely that a range of sources are generating and delivering sediment^15,17,44,80^. This also supports the hypothesis that mixing between a variety of sources^15,88^ occurs during various stages of transport; from the primary source to the sedimentary storage (loess). Fenn et al.^15^ argued that while the dominant zircon signature of loess reflected that of the Danube River, small changes could be seen between sites which they proposed were driven by smaller local sources and their contributions. These arguments are supported by Fig. 5D, where a slightly better fit is seen for Bulgaria (Slivata 1 and 2) and Romania (Balta Alba) and a better match for Serbia (Surduk 2) and Croatia (Erdut) with Alpine and Carpathian contributions (Fig. 5B, E).

It is important to note that none of these combinations explain the provenance of < 2 μm well. There are some similarities shown in Fig. 5A, B and E. Typically flysch (palaeo-accretionary wedge) has to be incorporated as an end-member to fit better with the fine grained loess component. Given that flysch represents already weathered and redeposited fine-grained material, it consequently has finer-grain sizes and more radiogenic Sr isotope values. It is also relatively easy to erode which is why it is a likely contributor and potentially a missing link in understanding sources of European loess. Alternatively, there might be an as yet unknown endmember composition (existing within the Danube’s drainage but no published data are available) capable of explaining loess isotopic signatures, though it is likely at the high Sr and low Nd end of isotopic signatures.

**Fig. 6:**
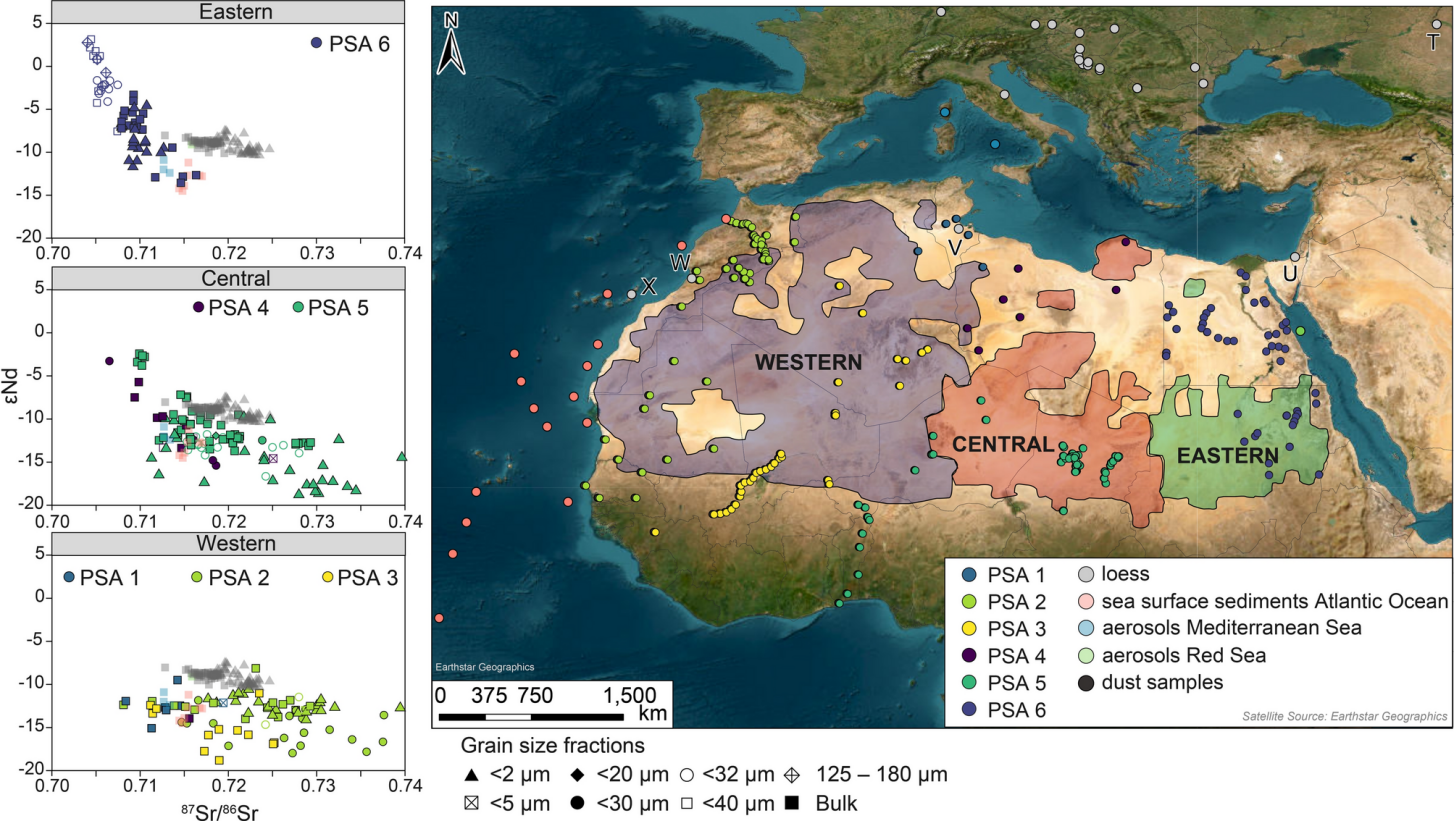
Sr–Nd isotopic composition of loess samples analysed in this study plotted with potential dust sources in North Africa and aerosol and sea-surface sediments. Areas are grouped by the Central, Eastern, and Western scheme introduced by Jewell et al. (2021) and colour coded according Potential Source Areas (PSA). Map shows the location of African sediment, soil, and bedrock, sea-surface, and aerosol samples. Capital letters refer to loess profiles T — Raigorod, U — Netivot, V — Matmata, W — Morocco, X — Fuerteventura. For source metadata see Supplementary Table 1. Map generated using ArcGIS PRO.

Meteorological observations document the annual Saharan dust fall over Europe during the instrumental period^21,99^. Moreover studies of modern dust trajectories, grain size, and grain shape suggest contributions of maximum 5–10%^14^ of Saharan dust to loess profiles, in particular during the interglacials^19,21,22^. The addition of clay fractions to the interglacial soils have been proposed to be as high as 20–40%^20^. Yet there is a growing body of geochemical literature which shows that the Sahara is in general an unlikely, at best a minimal, source of sediment for loess along the Danube River^14,15,24,100^ and other European loess profiles^101^. Whilst Saharan contributions to loess were shown to be improbable by Fenn et al. (2022), due to the large size of zircons (> 20 μm) analysed a Saharan addition to clay size fractions could not be excluded. Figure 6 (and Supplementary Fig. 4) shows ^87^Sr/^86^Sr and εNd for loess, North African sediment samples (coloured by country), aerosols collected from the Mediterranean Sea, and surface sediments collected from the Atlantic Ocean. Samples are grouped by the Eastern, Western, Central scheme proposed by Jewell et al. (2021) and separated by the grain size fraction. Figure 6A shows the Central area which corresponds to parts of Libya, Niger, and Chad (which combines Potential Source Area (PSA) 4 and PSA5) and have a fairly large range of isotopic values (^87^Sr/^86^Sr 0.7065–0.74 ; and εNd −2 to −18). Samples in this region also plot along a trendline; from more radiogenic Nd in Chad (εNd ~ -2) to the less radiogenic Nd isotopic values (and more radiogenic Sr) in Nigeria (εNd ~ -18). However most of the data from this region plots in a less radiogenic εNd space and below Danubian loess (−10 to −18). Source samples from Africa do however plot directly over the aerosol and sea-surface samples. Western source areas (Fig. 6B) represent Algeria, Morocco, Mauretania, parts of Niger, Senegal, and Tunisia (PSA1, PSA2, PSA3). These areas do not produce very much overlap with loess as almost all samples from these PSAs have less radiogenic εNd values (−10 to −18) and a fairly large range of strontium isotope ratios (^87^Sr/^86^Sr 0.707–0.74). However they provide a good match with aerosol sediments (sediment traps) from the Mediterranean Sea and the sea surface collected sediments from the Atlantic. Sediments from the Eastern Area (top panel Fig. 6; PSA6) show a range of values for ^87^Sr/^86^Sr (0.7041–0.7183) and εNd (3.3 to −13.6). Data plots along a trendline that represents three separate clusters of data; (1) radiogenic Nile sediments (which is consistent with the Nile’s volcanic source areas in Ethiopia); (2) Red Sea Hill that consists of Palaeozoic and Mesozoic clastic rocks^102^; and (3) sands from the Western Desert area of Sahara. Whilst most of the data does not overlap with loess the Western Deserts do match with aerosol and sea surface sediment.

**Fig. 7:**
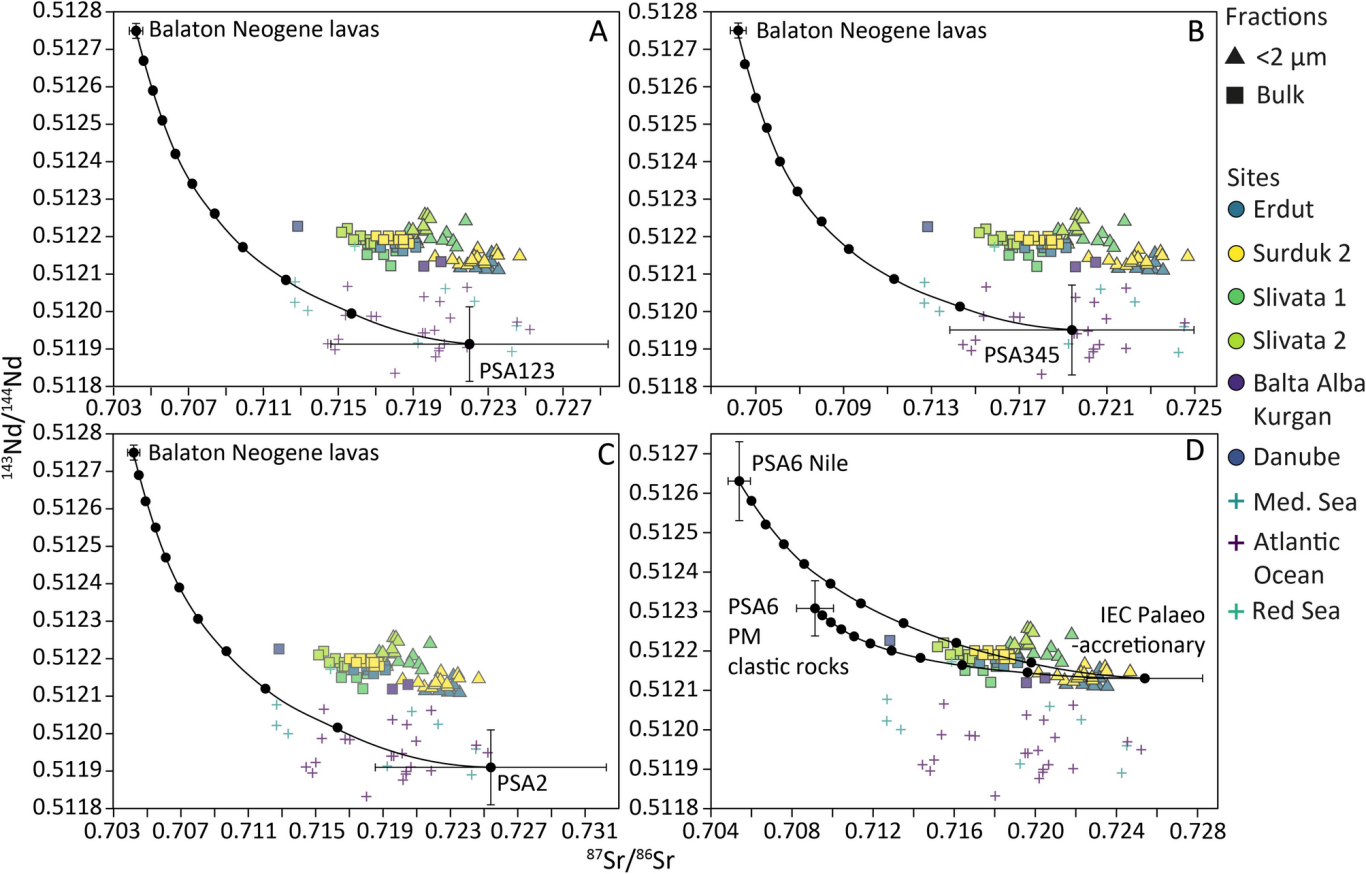
Mixing lines between averaged most common dust emitting areas PSA 1, PSA2, PSA 3, PSA4, PSA5 and Neogene volcanic rocks in Balaton lake area^95^ (including the standard deviation of the dataset); **D** PSA6 (split into Nile and Palaeozoic and Mesozoic clastic rocks samples) mixed with European flysch. PSA mean points have been calculated only from datasets that have corresponding elemental data (Supplementary Fig. 3). In all cases data from dust from Mediterranean Sea and Red Sea aerosols, and Atlantic Ocean surface sediments^105,113^ are also included. Mixing lines calculated following Faure (2001).

Figure 6 shows that most of the data from Sahara does not overlap with loess but it could offer a potential good mixing end member especially for the “crustal” (less radiogenic Nd and more radiogenic Sr) values which are not very abundant in the European bedrock dataset (this could of course be bias in the available data). The particularly active dust areas in North Africa (Fig. 6 and Supplementary Fig. 4) have long been studied using not just modern day trajectory tracing but also geochemical and mineralogical approaches^21,103–107^. Varga et al. (2013) proposed three types of modern storm tracks (types 1, 2, and 3) that may contribute dust particles to loess sequences along the Danube. The most dominant (66% of the time) Type-1 encompasses PSAs 1, 3, and 4. Type-2 originates from PSAs 3, 4, and 5 and occurs 25% of the time. The least dominant, Type-3 (9%), is sourced from PSA2 (Supplementary Fig. 4). These were used in this study to test their potential contributions to Danubian loess (Fig. 7) on their own but also mixed with the radiogenic bedrock end member from the Pannonian Basin (Balaton Neogene volcanic) as these types of contributions are mostly missing from North Africa. 

Overall Fig. 7 shows that North African samples located in regions where dust storm tracks originate from do not generate mixing lines that can be used to explain Sr and Nd isotopic composition of loess, even when the uncertainties in the dataset are taken into consideration. Figure 6 already showed that most of the variability in Saharan datasets come from Sr isotopic composition, which is not surprising given the variability in grain size, weathering patterns, and clay content between these samples. However, within Nd values (a more reliable provenance indicator of the two) these source regions are well constrained. The mixing lines attribute aerosol dust collected in the Mediterranean Sea and sea-surface sediment from the Atlantic Ocean to the African sourcesand demonstrate the applicability of Sr–Nd as a provenance tool. The mixing between Type-1 (a combination of PSA1, PSA2, PSA3; Fig. 7A), Type-2 (a combination of PSA3, PSA4, PSA5; Fig. 7B), and Type-3 (PSA3; Fig. 7C) dust storm sources plotted against Balaton lava composition (Fig. 7A) shows that none of these explain loess Sr and Nd isotopic values. The only mixing model that explains loess isotopic values and uses a potential North African source combines Nile sediments (PSA6; Fig. 7D) and the paleo-accretionary wedge from the Eastern Carpathians. However samples from PSA6-Nile come from Egypt and Sudan; areas that do not fit reconstructed dust transport trajectories^18,108,109^. Moreover a close inspection of this mixing line shows that it fits better (i.e. explains better) loess bulk sample composition rather than fine grained dust material (contributions of 20% and 10–5% respectively). This is not an expected relationship for a far travelled dust as it is argued that Saharan sources would contribute predominantly fine silt and clay grain sizes. Consequently Saharan signal should be stronger in the < 2 µm fractions. Finally, the timing and volumes of sediment also must be considered. The studies that support Saharan contributions into Danubian loess evoke modern dust storms tracks as a transport mechanism ^18,21^ and argue for particularly significant contributions during interglacial periods^20^. The samples analysed here show that in any case the Last Glacial Maximum loess, rather than interglacial palaeosol samples (tested range includes the Holocene, Marine Isotope Stage 5, and Marine Isotope Stage 7; Supplementary Fig. 5), is a closer match to Saharan sediment, contradicting previous suggestions. Figure 6 also demonstrates Danubian loess isotopic signatures can be very easily explained using a multitude of European bedrock end-members. Consequently North African dust contributions are not only a bad geochemical fit but are also not needed to explain signatures measured in loess and palaeosols, supporting a recent clay mineralogy study^100^. By comparison Sr and Nd isotopic signatures of aerosols and sediments collected over the Mediterranean Sea and Atlantic Ocean are closely aligned with North African sources supporting northward transport of dust from potential source areas^110,111^. The results of this study do not imply that dust-bearing storms originating from North Africa were absent during loess deposition; rather, they did not serve as the primary transport mechanism for the sediments forming loess deposits. While this analysis does not enable the reconstruction of palaeowind directions for Danubian loess profiles, the findings support the suppression of southwesterly winds and a southward shift of westerly winds driven by the expansion of northern European ice masses^17,26,27,100^. Consequently, southwesterly winds originating in and passing over North Africa predominantly reached southern Europe^111,112^, with limited transport beyond. Minimal contributions from the Sahara further suggest that northern Saharan winds were not directed toward the Danubian Basin.

In summary based on the Sr–Nd isotopic composition of loess deposits along the Danube River compared to extensive loess, bedrock, dust, and aerosol datasets this study demonstrates that sediment for loess deposits was sourced from surrounding mountain belts, i.e. the Alps, Balkan Mountains, Bohemian Massif, Carpathians, Dinarides, and Pannonian Basin. A semi-quantitative approach goes a step further from previous studies demonstrating that the majority of the sediment (50–90%) comes from rocks with low radiogenic Nd values, such as the metamorphic basement and paleo-accretional sedimentary rocks (flysch) of the Eastern Carpathians or as of yet unidentified rocks similar in composition to flysch, most likely the Neogene fine-grained infill of the Transylvanian, Pannonian and other sedimentary basins in the area. These rocks are abundant across the Carpathians and Alps but largely underrepresented in term of geochemical datasets available. The potential candidates for the second end-member with more radiogenic Nd signatures are plentiful along the Danube River and many of them produce a good match for signatures measured in loess. This work also demonstrates that North African dust regions are not necessary to explain signatures of Danubian loess–palaeosol profiles. North African dust exhibits mixing models that rarely overlap with loess signatures. Even in instances where overlaps occur, they are typically associated with regions like Egypt and Sudan, which do not support dust trajectories toward the Danubian Basin. Moreover, these overlaps better explain bulk sediments rather than fine-grained components and align more closely with loess than with palaeosol, contradicting earlier studies^20,114^. The findings suggest that the Sahara contributed negligibly, in terms of volume, to the material forming Danubian loess. Instead, the Sahara emerges as a clear source of dust deposited over the Mediterranean Sea. The results presented here suggest predominantly westerly^11,26,29^ wind patterns during glacial periods in the Danubian Basin.

## Methods

### Sample collection and sediment preparation.

Four loess–palaeosol profiles along the middle and lower reaches of the Danube River (Fig. 1) in Croatia, Serbia, North Bulgaria, and Romania were investigated. All have stratigraphy, lithology, and chronology results already published^50–53^. Pilot samples from Balta Alba Kurgan (Romania) capture the dust deposition during the Holocene. Surduk (Serbia), and Slivata (Bulgaria) span the last glacial-interglacial cycle, whilst the Erdut (Croatia) records cover the penultimate glacial-interglacial cycle (Supplementary Table 2). A total of 47 samples were analysed, including 13 from Erdut, 15 from Surduk, 17 from Slivata, and 2 from Balta Alba Kurgan. Additionally, a sample was collected from modern exposed sandy-silty Danubian alluvium. For the samples from Balta Alba Kurgan and the Danube sample only bulk material was analysed. The remaining samples had two sediment fractions analysed, bulk and < 2 µm. This approach accounts for the grain size effect on Sr isotopic ratios^41,64,115^ and encompasses the dominant grain size fraction of modern dust derived from Sahara (1–2.5 μm^108^) though larger grains are observed. To separate the < 2 µm fraction, samples were settled in distilled water in accordance with Stokes Law.

### Sr–Nd analysis

Nd and Sr analysis were conducted at the Geochronology and Tracers Facility at the British Geological Survey, following methods published in Fielding et al. (2016) and Bird et al. (2020)^41,102^. Powdered 150–200 mg of each sample was weighed into 15 ml Savillex PFA vials and leached in 5 ml of 10% acetic acid at 60 °C for 2 h to remove carbonate. After discarding the leachate, the samples were washed and centrifuged twice in Milli-Q water, dried and reweighed. Subsequently, 1–2 ml of 2 × PFA-distilled 16 M HNO3 and 5–6 ml of 29 M HF were added to the samples, which were then evaporated to dryness at approximately 105 °C overnight. The process continued with the addition of 1–2 ml of HNO3, followed by drying on a hotplate. To convert the samples to chloride form, 10 ml of PFA-distilled HCl was introduced. For primary column chemistry, the samples were dissolved in approximately 2 ml of calibrated 2.5 M HCl and centrifuged to remove any residues.

Approximately 1 ml of dissolved sample was pipetted onto quartz-glass columns containing 4 ml of AG50 × 8 cation exchange resin. Matrix elements were washed off the column using 48 ml of calibrated 2.5 M HCl, and discarded. Sr was collected in 12 ml of 2.5 M HCl, and evaporated to dryness. A bulk rare-earth element (REE) fraction was collected in 15 ml of 6 M HCl and evaporated to dryness. Nd was separated from the bulk REE fraction using 2 ml of EICHROM LN-SPEC ion exchange resin packed into 10 ml Biorad Poly-Prep columns. The bulk REE fraction was dissolved in 300 μl of 0.2 M HCl and loaded onto the columns. La, Ce and Pr were eluted using a total of 16 ml of 0.2 M HCl. Nd was collected in 4 ml of 0.3 M HCl.

Nd fractions were analysed on Thermo Scientific Neptune Plus and Triton mass spectrometers. On the Neptune, samples were dissolved in 1 ml of 2% HNO_3_ prior to analysis in static multicollection mode. On the Triton, Nd fractions were loaded onto one side of an outgassed double Re filament assembly using dilute HCl and analysed in multi-dynamic mode. Data are normalised to ^146^Nd/^144^Nd = 0.7219. Across the time of analysis, 28 analyses of the JNd-i reference material^116^ on the Triton gave a mean value of 0.512104 ± 0.000007 (1-sigma); 287 analyses of the JNd-i reference material^116^ on the Neptune gave a mean value of 0.512071 ± 0.000015 (1-sigma). Results are quoted relative to a value of 0.512115 for this standard. Six analyses of the BCR-2 rock standard run with the samples gave a value of 0.512637 ± 0.000009 (1-sigma).^143^Nd/^144^Nd ratios in this study are also reported as εNd calculated using the present-day chondritic uniform reservoir (CHUR) value of 0.512630 ± 0.000011^117^.

Sr fractions were loaded onto outgassed single Re filaments using a TaO activator solution and analysed in a Thermo-Electron Triton mass spectrometer in multi-dynamic mode. Data are normalised to ^86^Sr/^88^Sr = 0.1194. Across the period of analysis, 270 analyses of the NBS987 reference material gave a mean value of 0.710260 ± 0.000006 (1-sigma). Sample data are normalised using a preferred value of 0.710250 for this standard. All data for samples, including element concentrations and ratios, can be found in Supplementary Table 3.

### Supporting datasets and data visualisation

To investigate loess sources and calculate mixing lines between potential sources, Sr–Nd isotope datasets were collected from published literature, which amounts to over 900 samples (Supplementary Tables 1, 2, and 3). This supporting dataset covers a range of geomorphic settings such as loess, fluvial, alluvial, desert, and bedrock. End-members were primarily grouped by location. Each end-member typically represented several values and therefore averages standard deviations were calculated prior to plotting. Mixing between end-members to produce mixing hyperbolae were calculated following equations outlined in Faure^118^. Dataset used to calculate end-member mixing in Fig. 7 is smaller than the one presented in Fig. 6 (Supplementary Fig. 2) as not all data points had corresponding elemental composition values.

## Supplementary Information

Supplementary Information 1.

Supplementary Information 2.

Supplementary Information 3.

Supplementary Information 4.

## Data Availability

All data generated or analysed during this study are included in this published article and its supplementary information files.
