# Supplementary material for: Provenance of late Pleistocene loess in central and eastern Europe: isotopic evidence for dominant local sediment sources
Source: Sci Rep. 2025 Jan 10;15:1624. doi: 10.1038/s41598-024-83698-5 (PMC11724038; doi:10.1038/s41598-024-83698-5)
Supplement: Supplementary file 4 — Supplementary Information 4. [file 41598_2024_83698_MOESM4_ESM.docx]

Supporting Information for

**Provenance of late Pleistocene loess in Central and Eastern Europe: isotopic evidence for dominant local sediment sources**

*Fenn, K., Millar, I. L., Bird, A., Veres, D., Wagner, Doris*

# **Supplementary Figure 1**


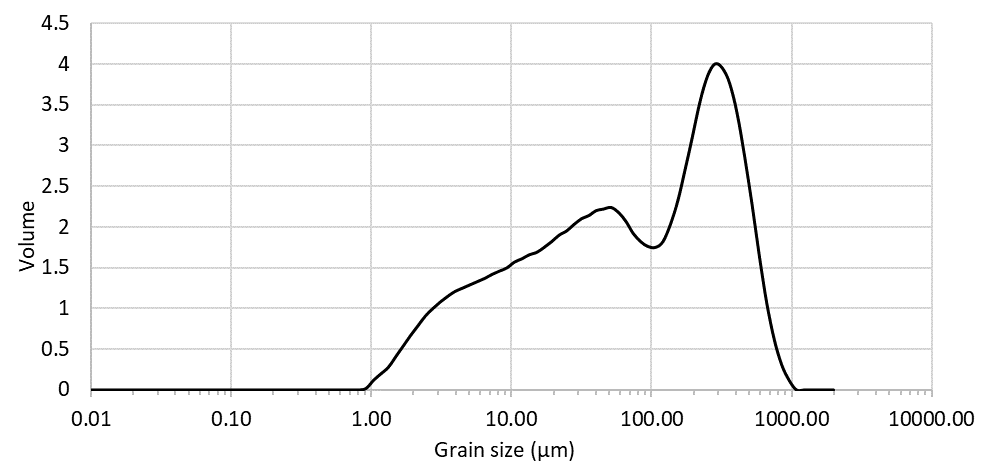


**Figure S1.** Grain size distribution for the Danube’s alluvium sample. Analysis conducted using a Bettersizer with Mie optical theory setting. Sediment was dispersed with Calgon prior to the analysis.

# **Supplementary Figure 2**


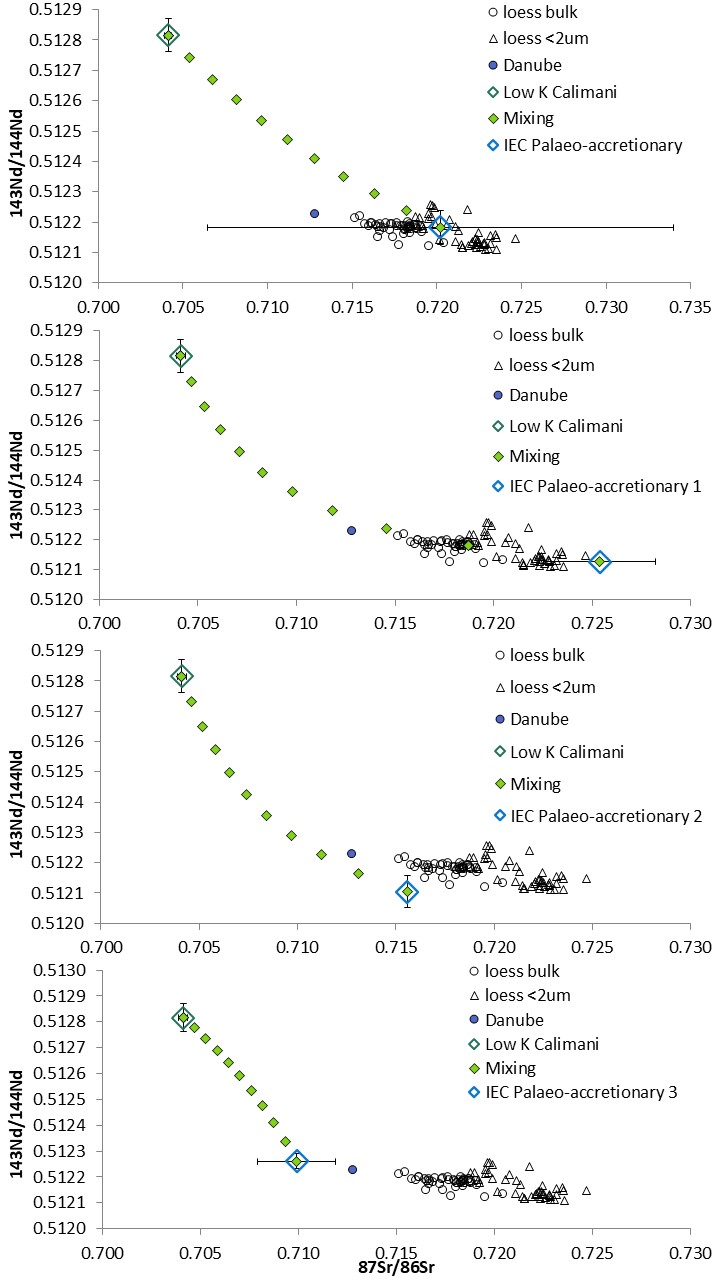


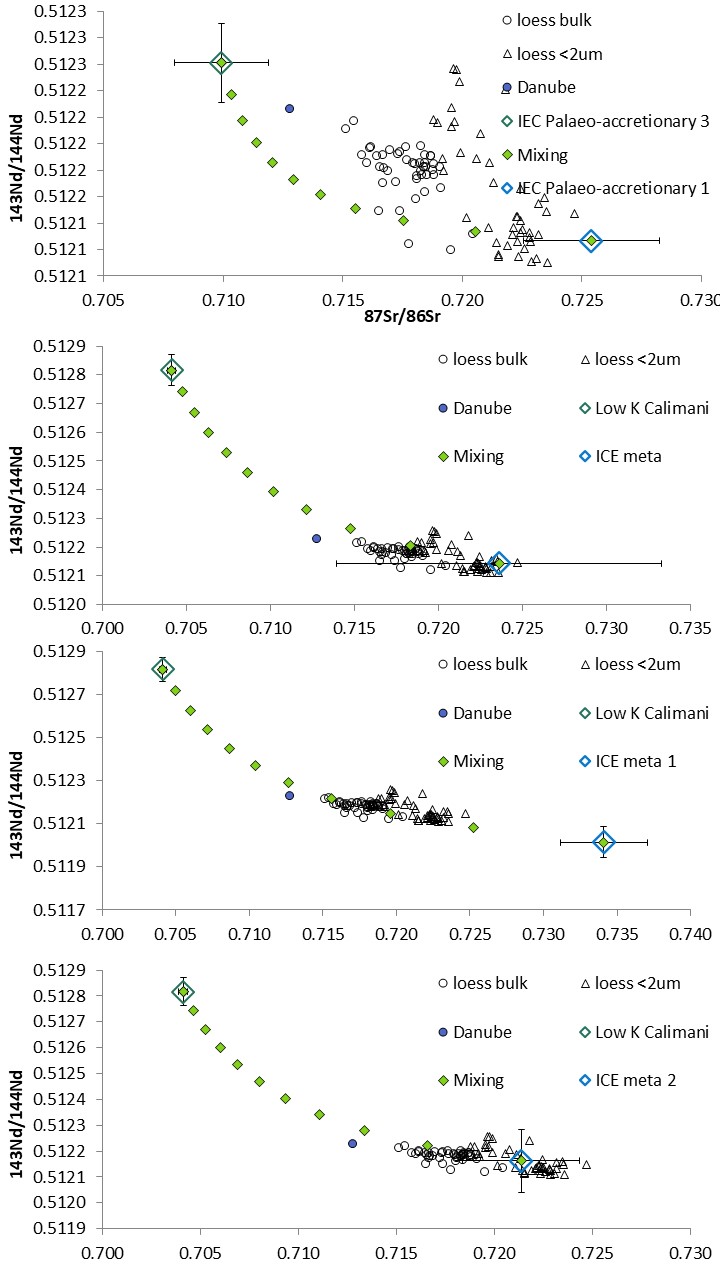

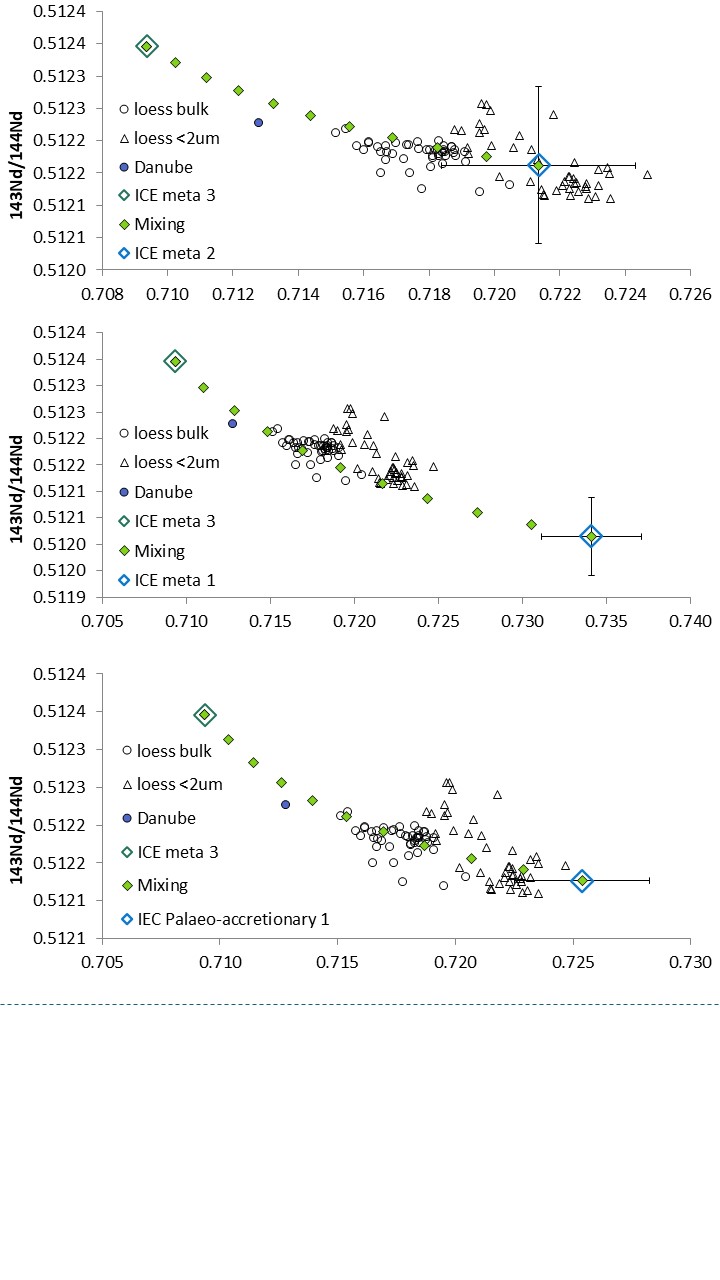

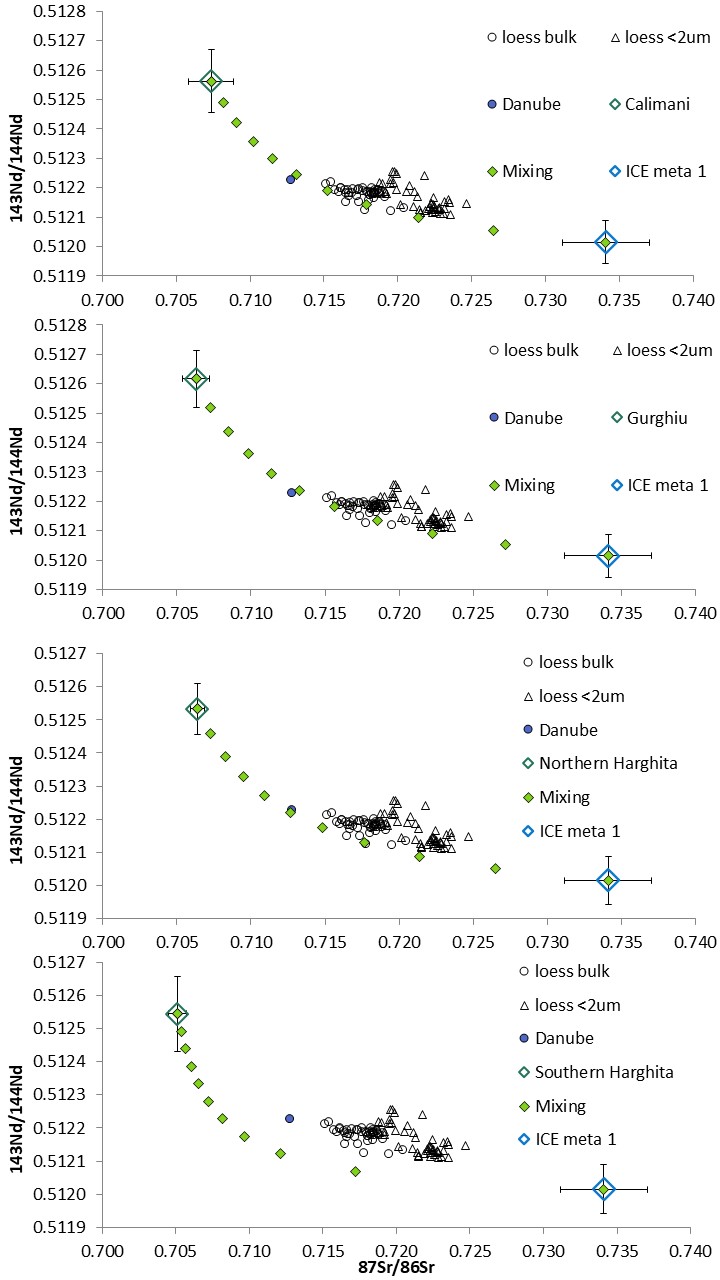

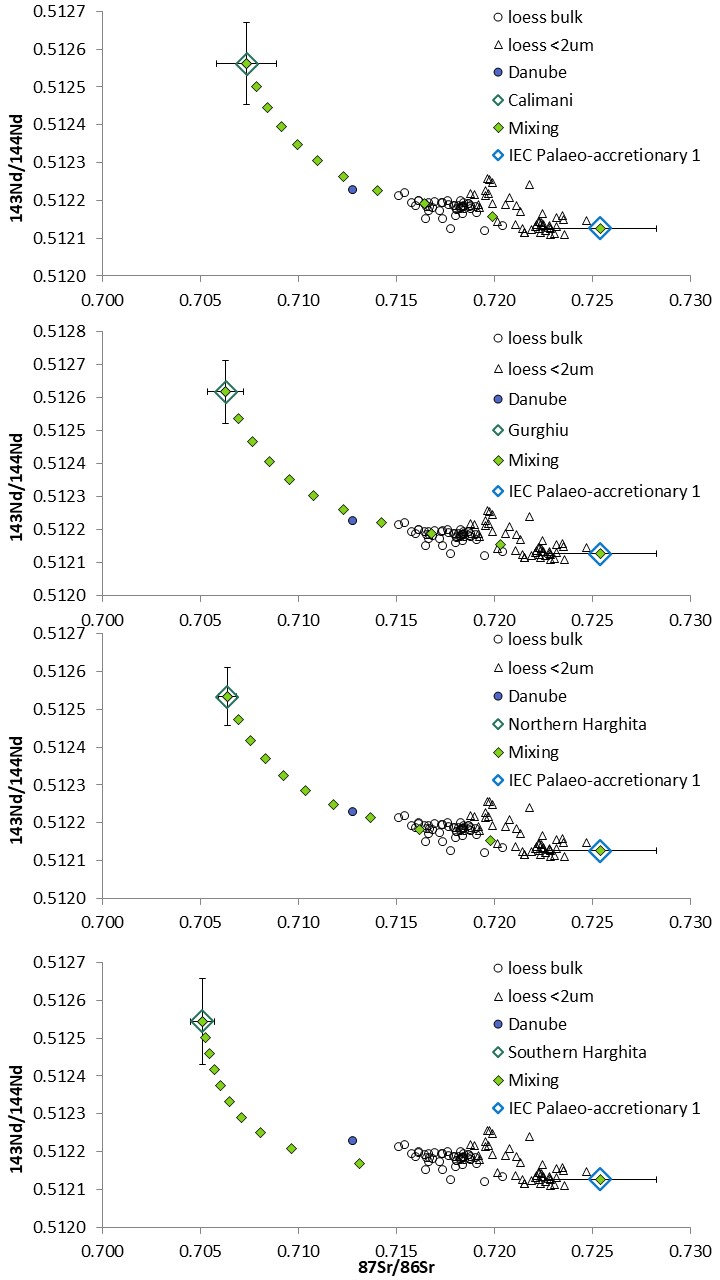

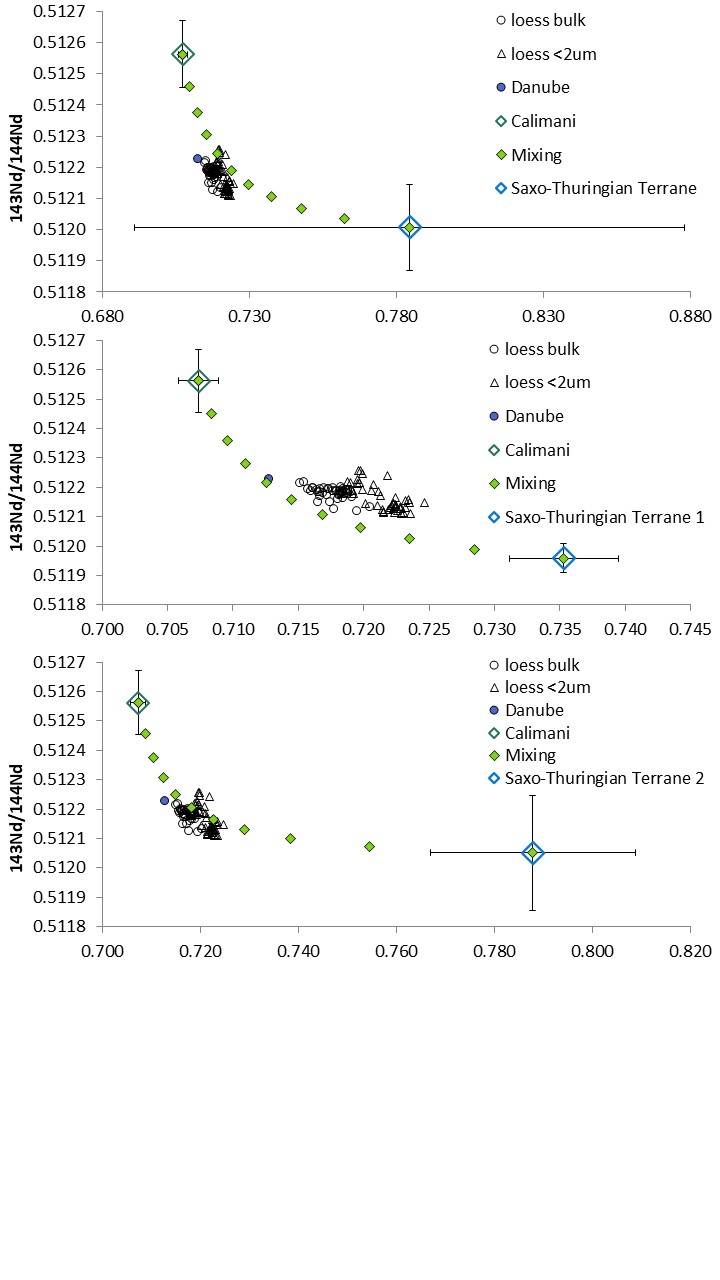

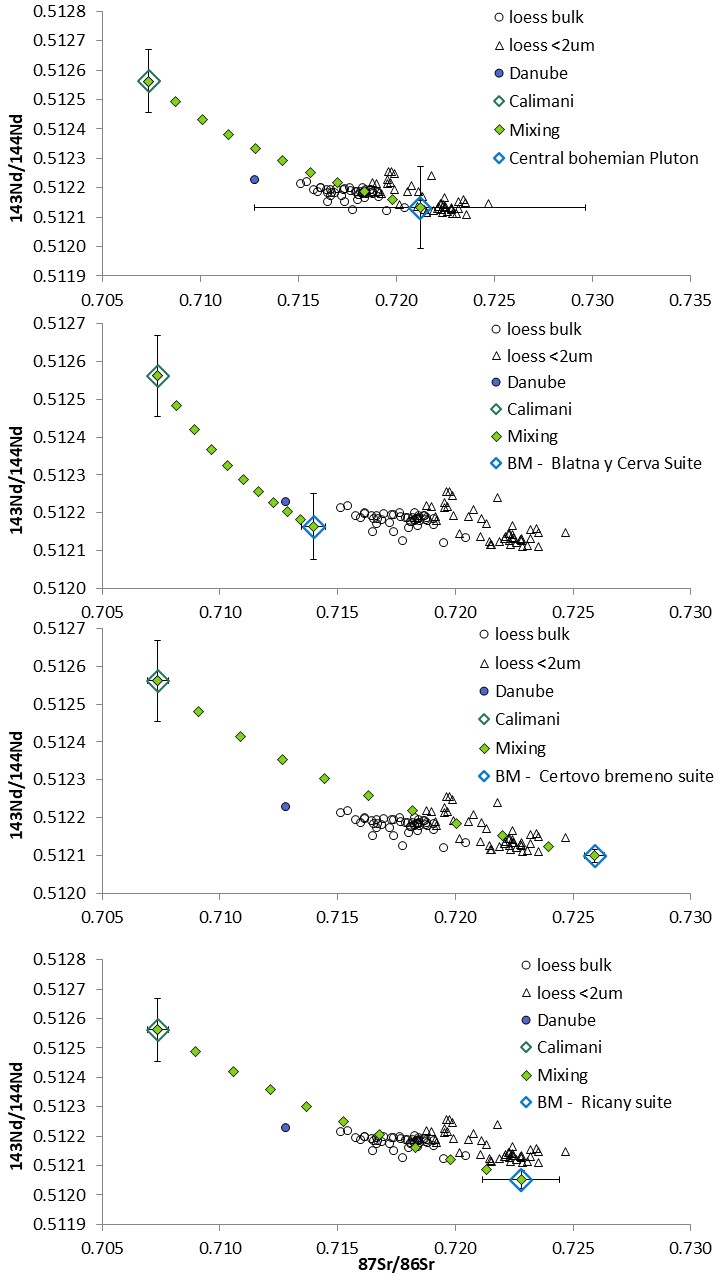

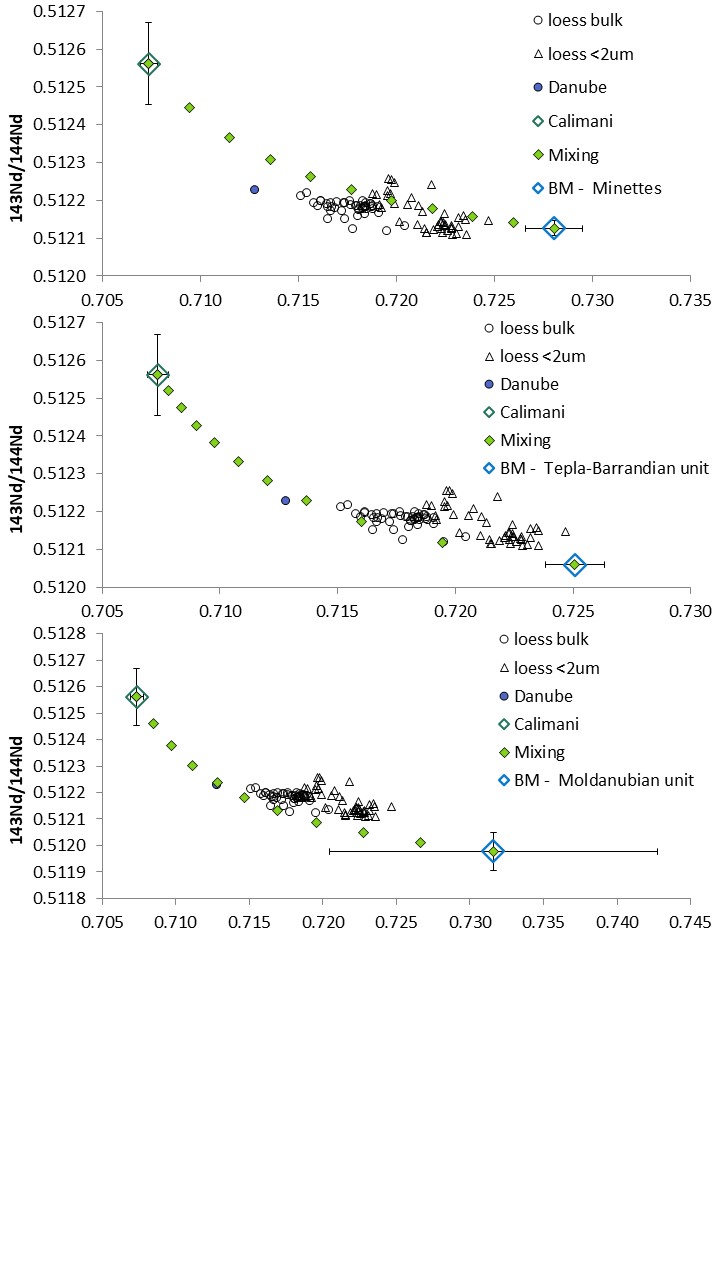

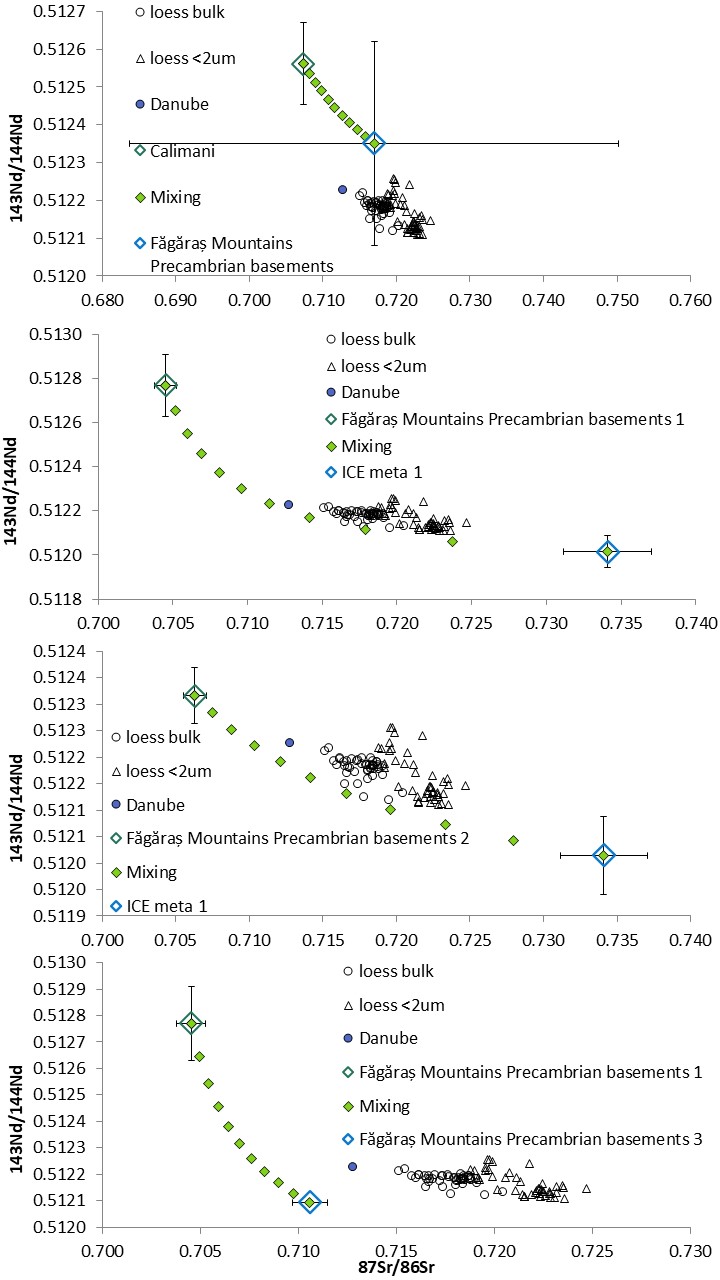

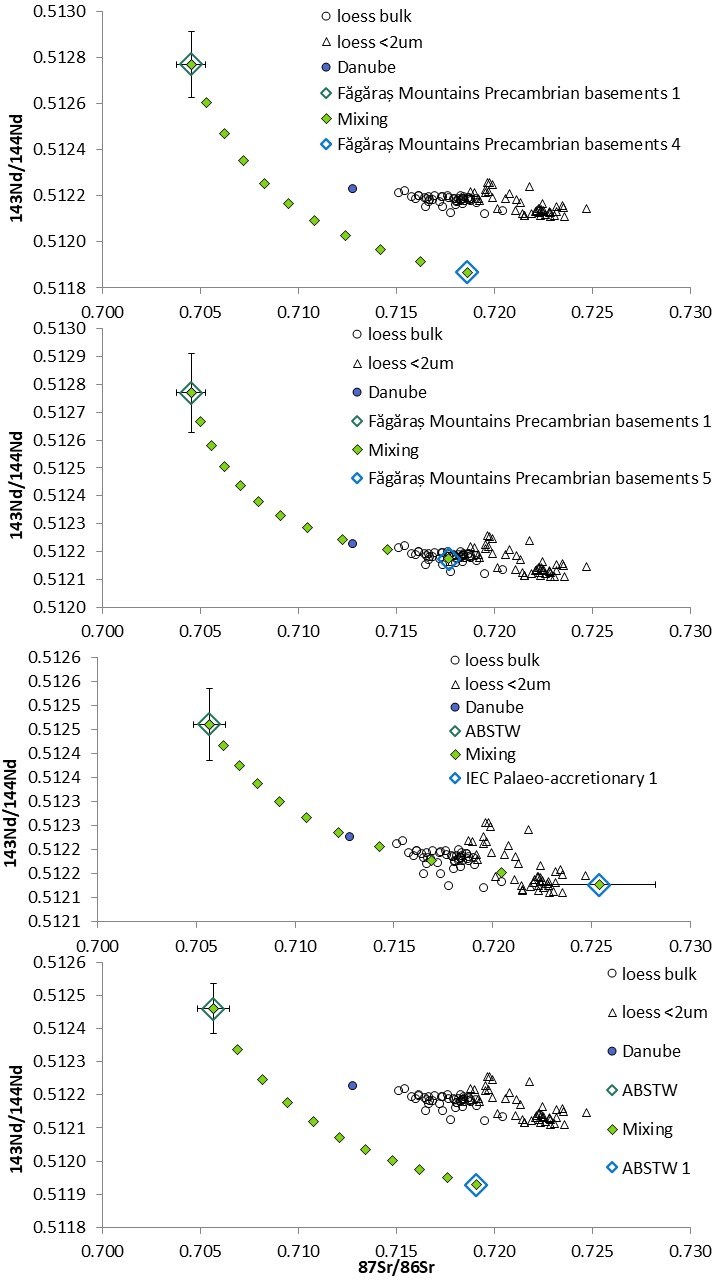

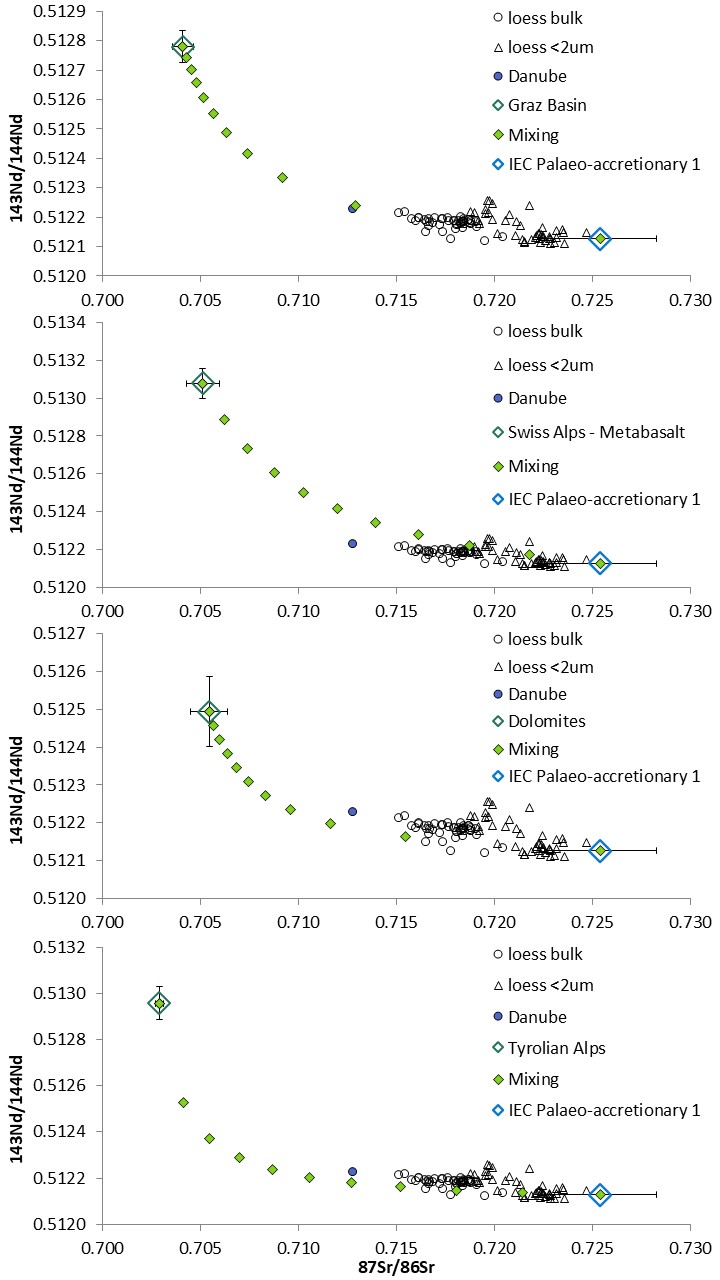

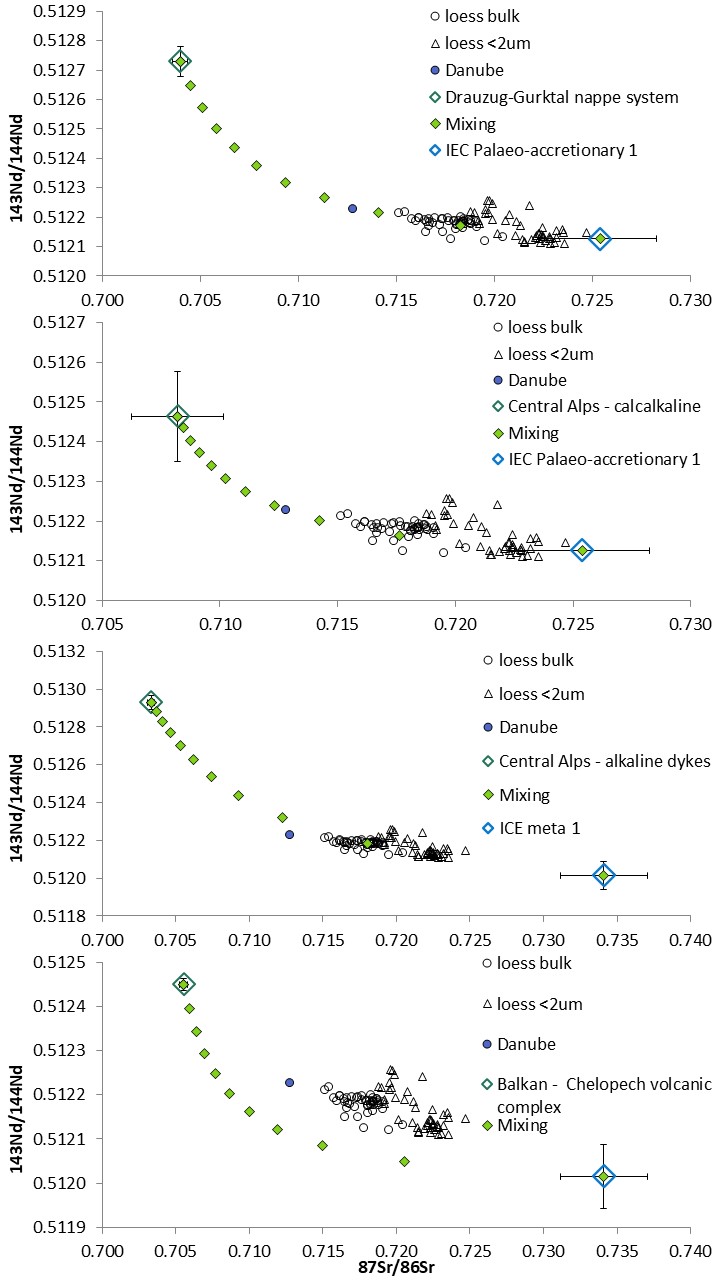

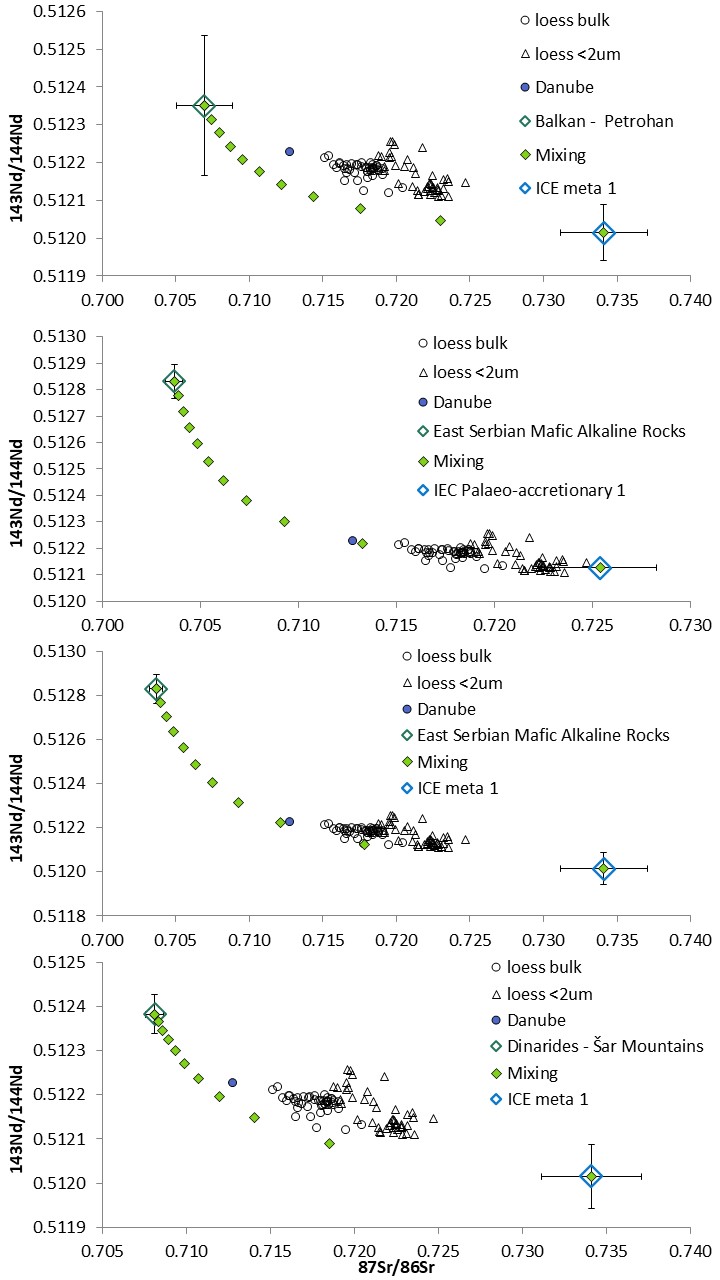

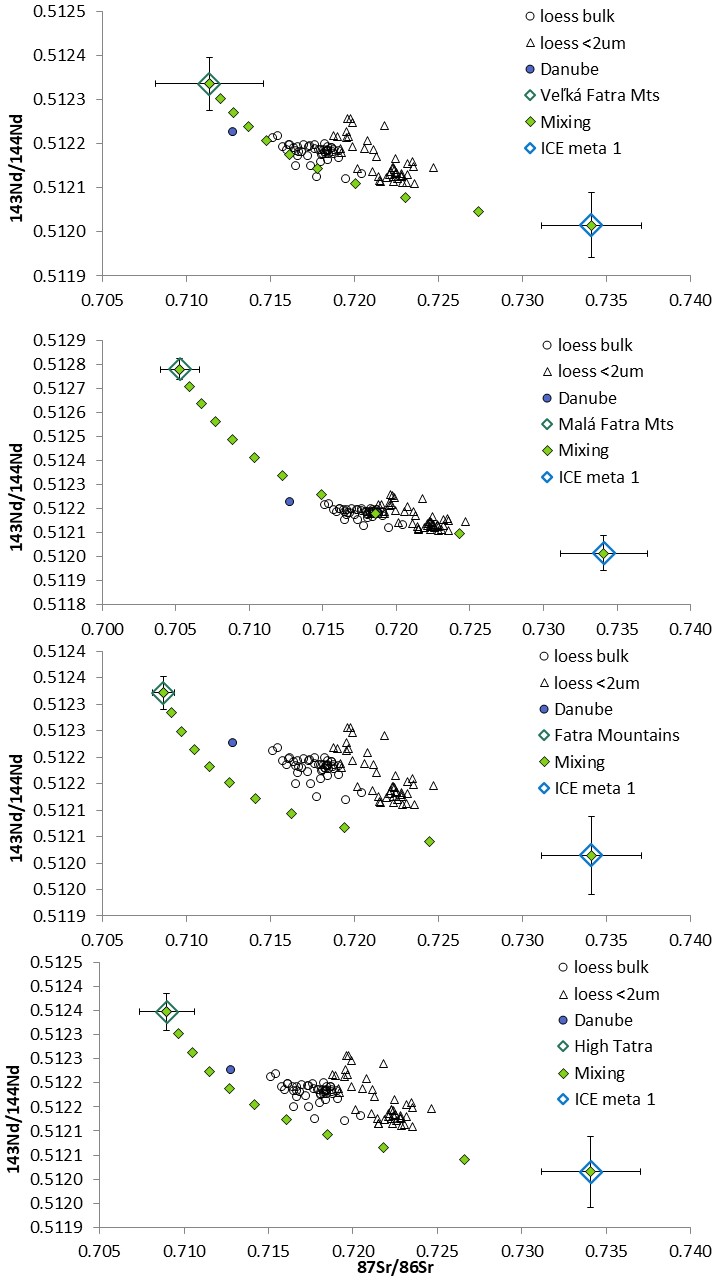

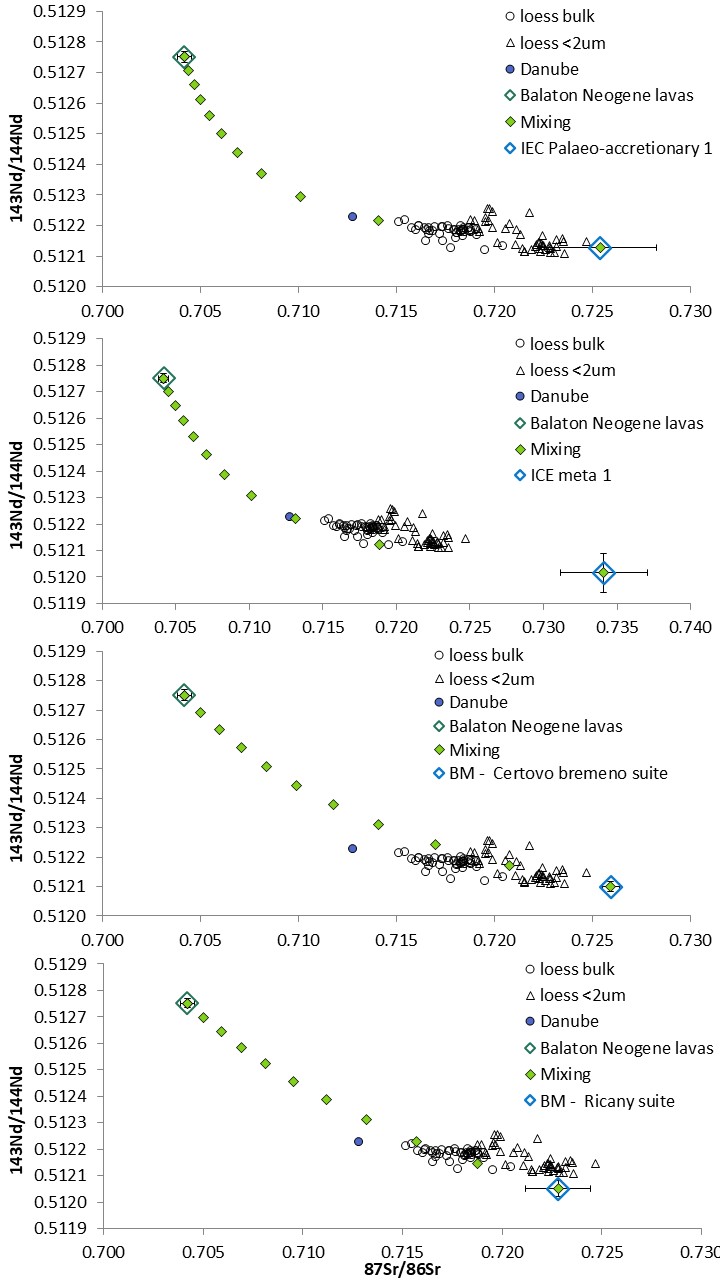

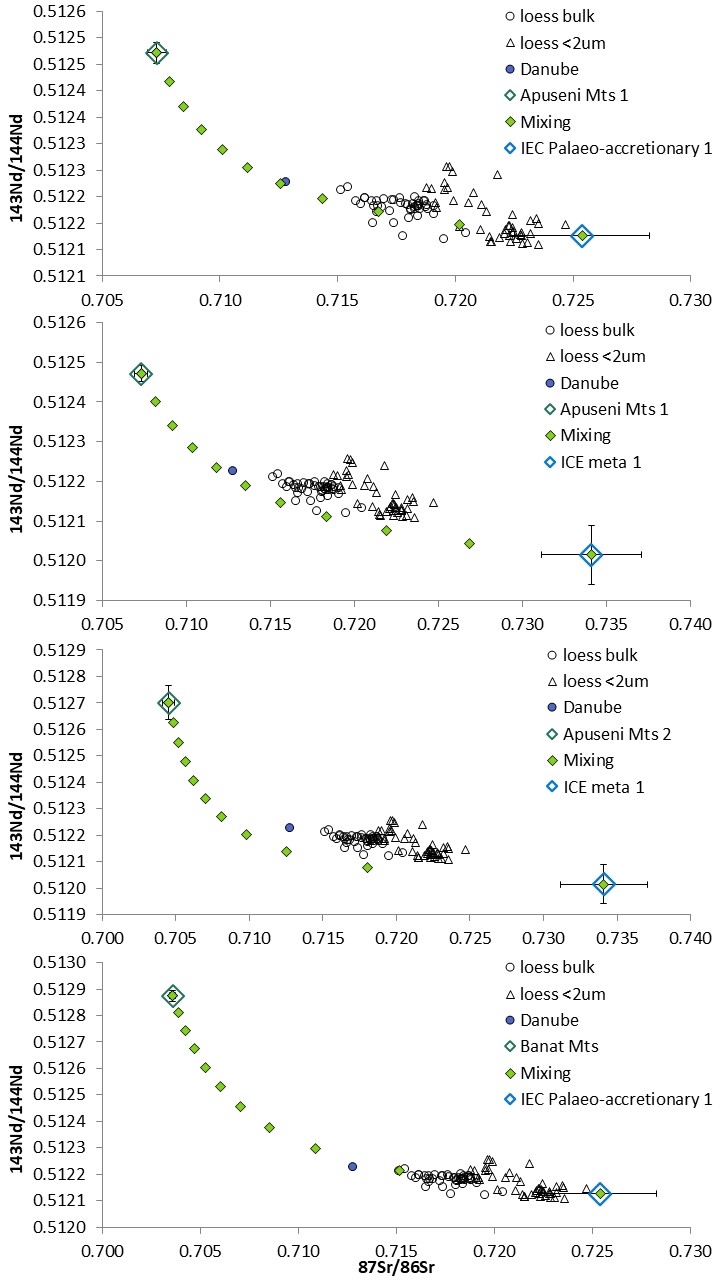

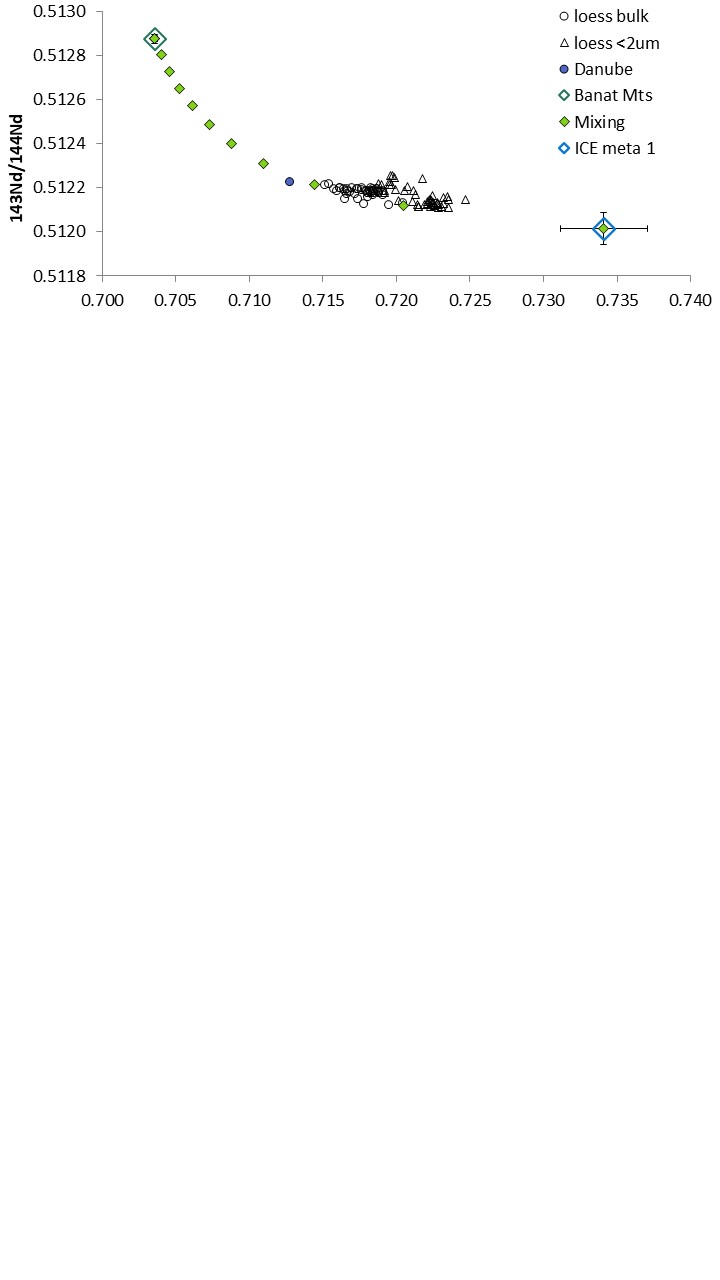


**Figure S2.** Mixing lines between averaged values of bedrock end-members for a range of explored combinations.

# **Supplementary Figure 3**


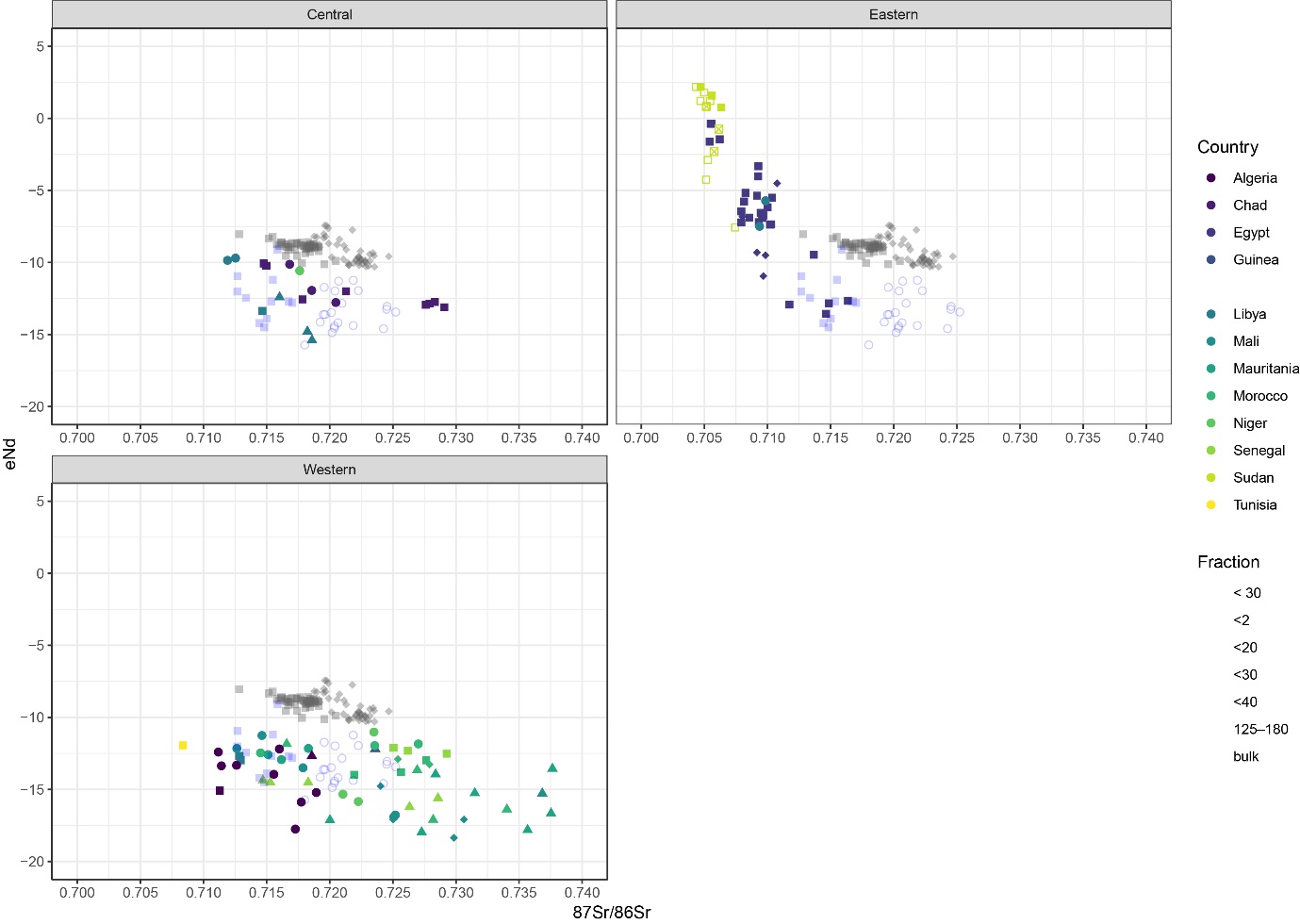


**Figure S3.** Sr-Nd isotopic composition of loess samples analysed in this study plotted with potential dust sources in North Africa and aerosol samples from the Mediterranean Sea and the Atlantic Ocean. This is a figure similar to Figure 6 from the manuscript but only contains samples used in calculated mixing models between end members (data that has corresponding Sr and Nd elemental data). Samples are separated by various grain size fractions. Areas are grouped by the Central, Eastern, and Western scheme introduced by Jewell et al. (2021). Additionally, dust source data is colour coded by country.

# **Supplementary Figure 4**


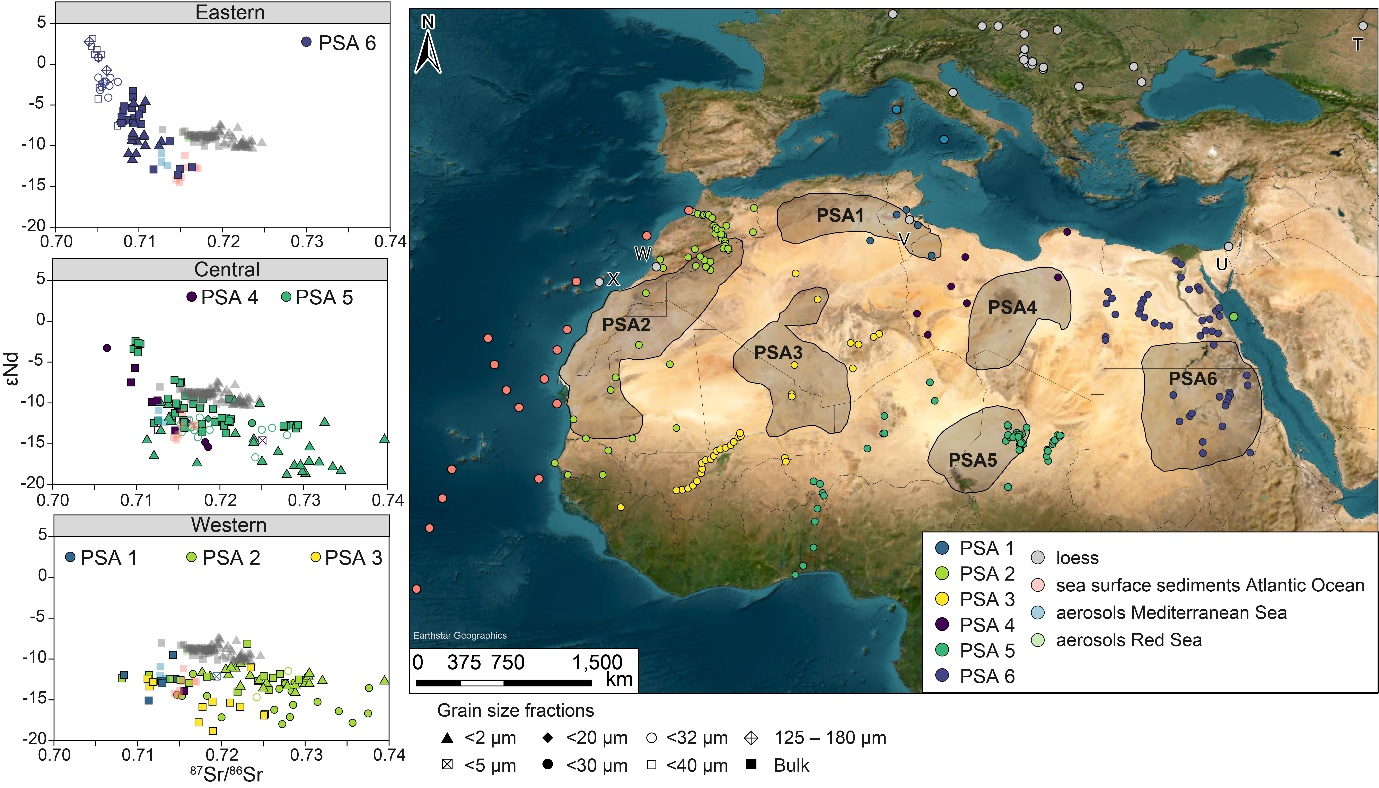


**Figure S4.** Sr-Nd isotopic composition of loess samples analysed in this study plotted with potential dust sources (PSA) in North Africa and aerosol and sea-surface sediments. Dust sources data is colour coded by country.

# **Supplementary Figure 5**

**
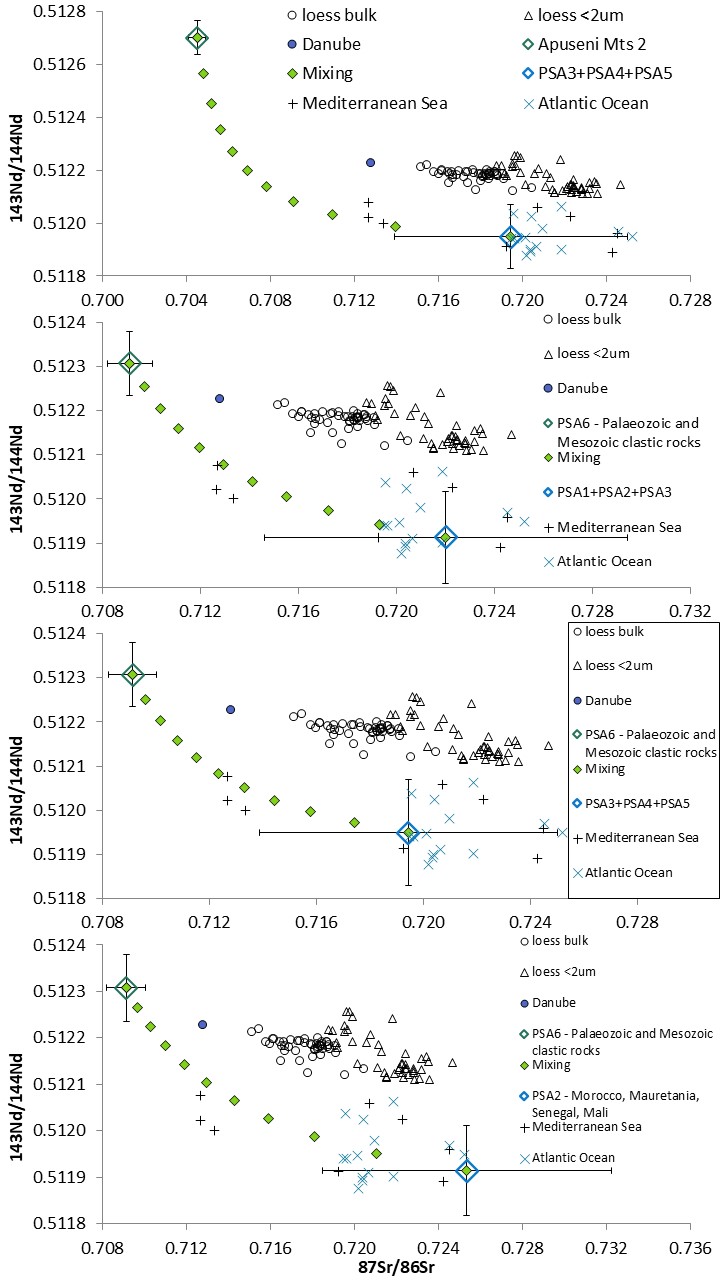
**


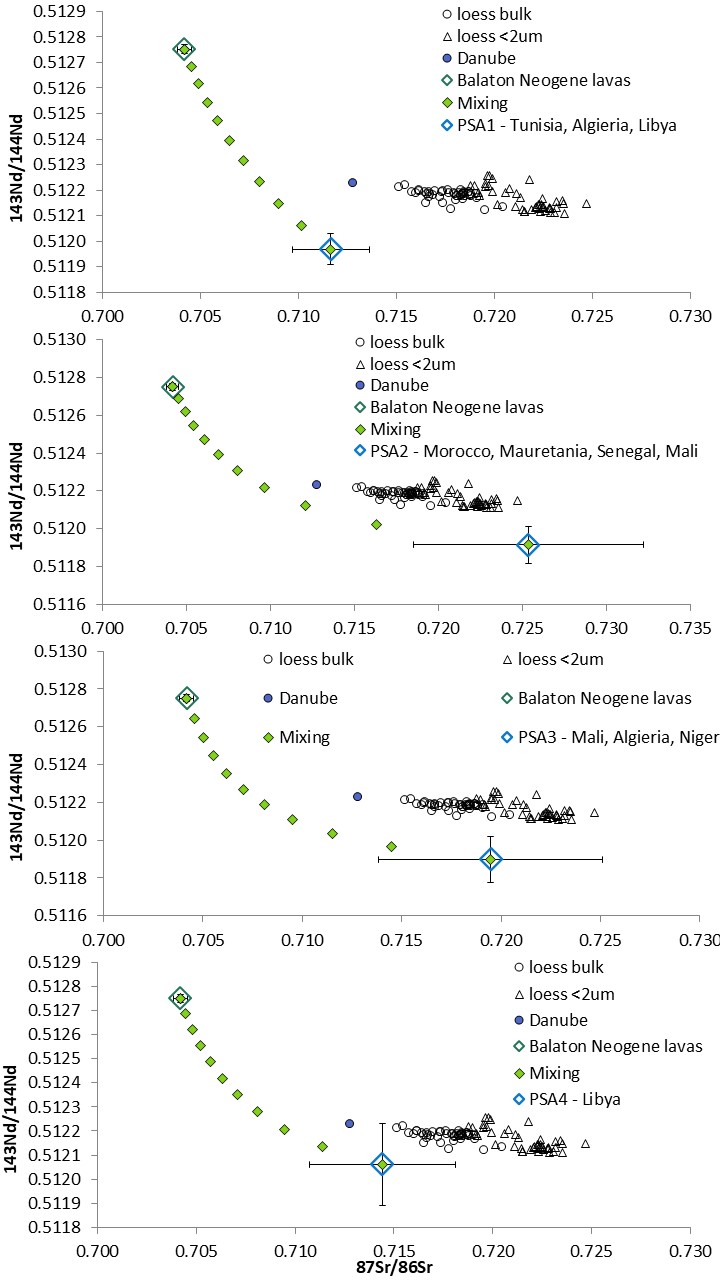

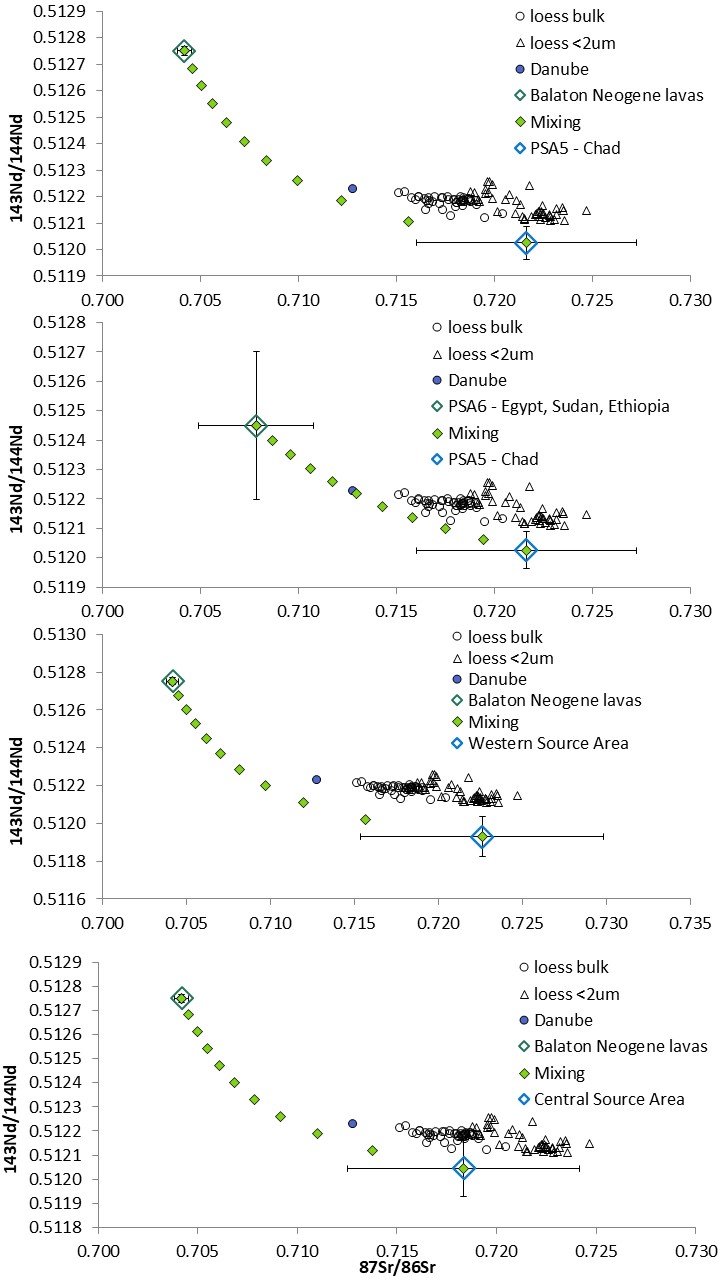

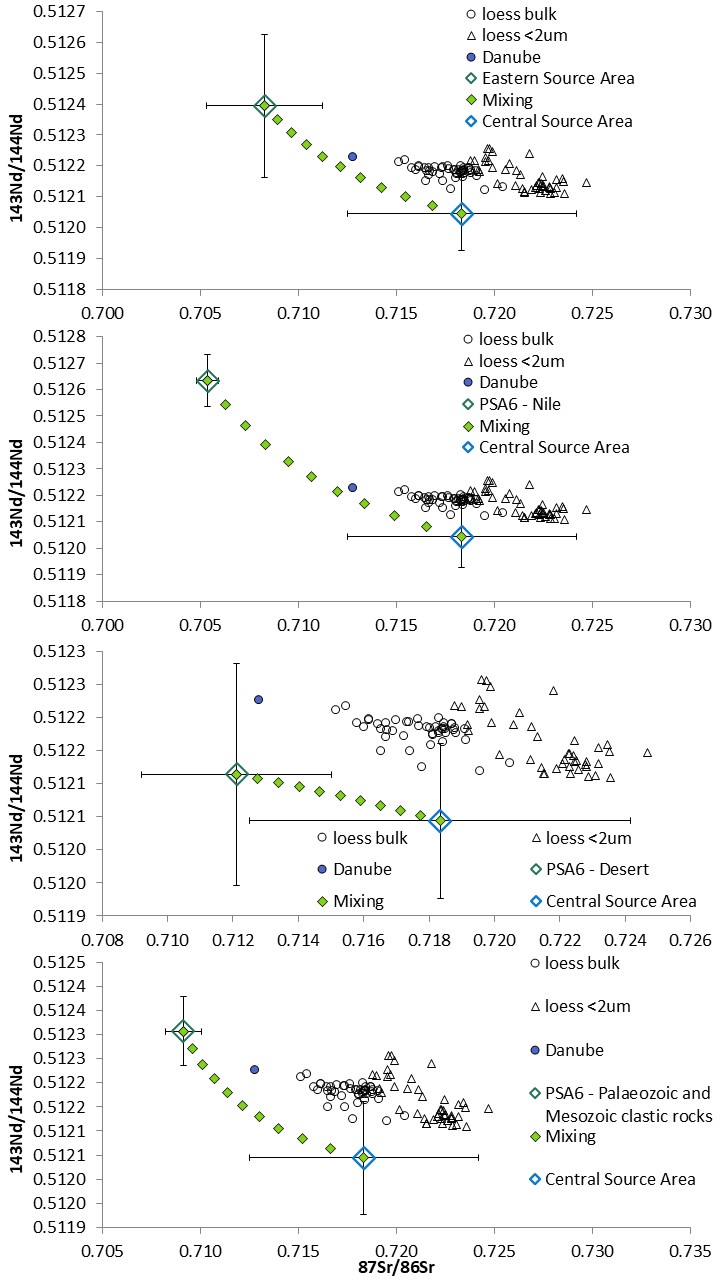

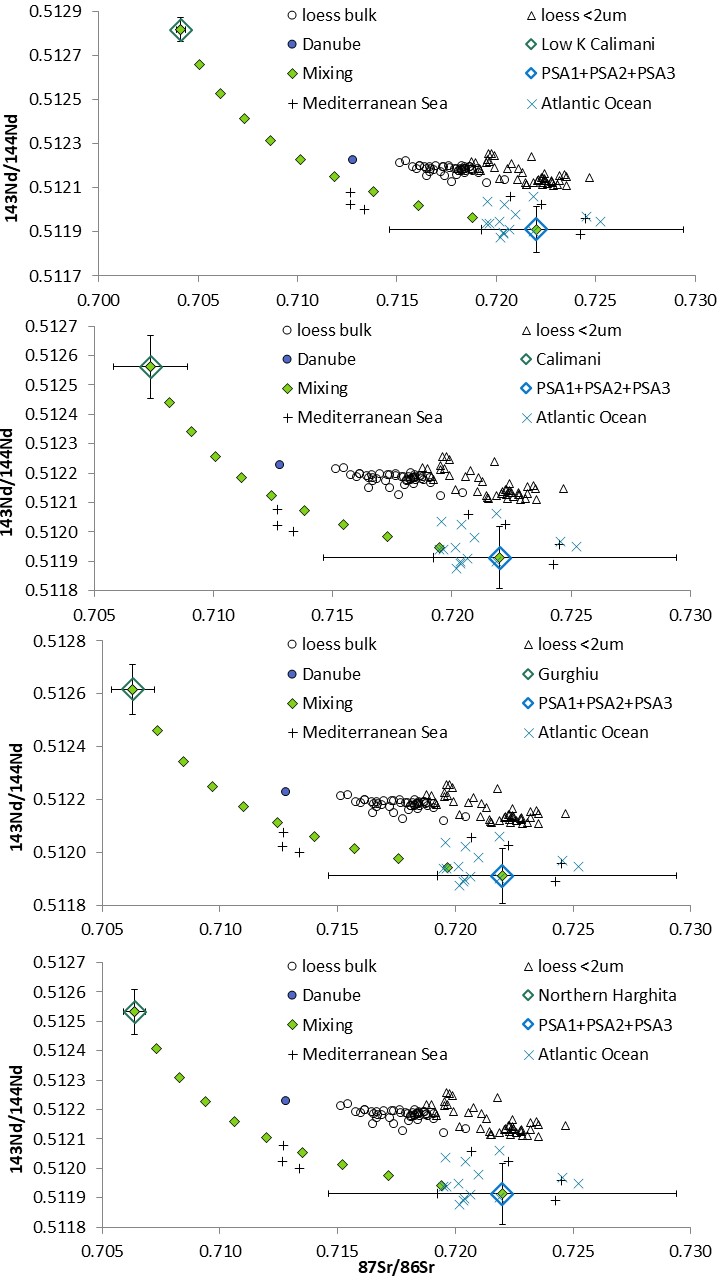

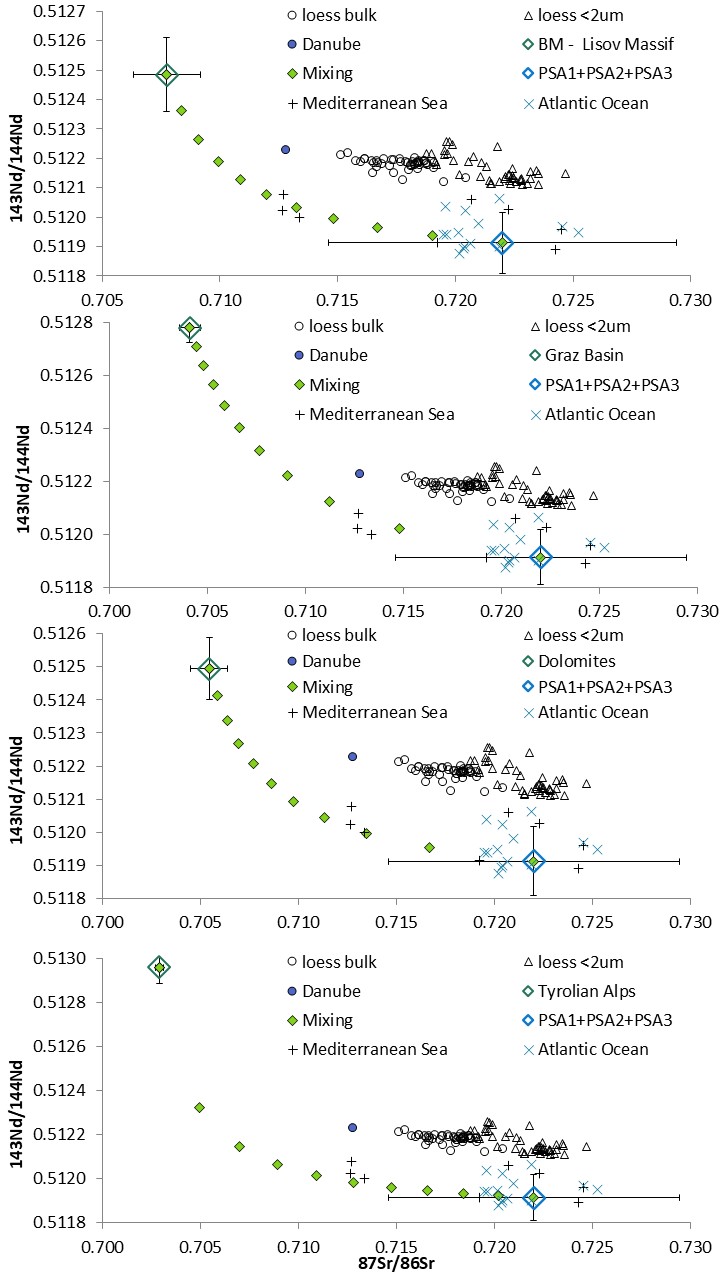

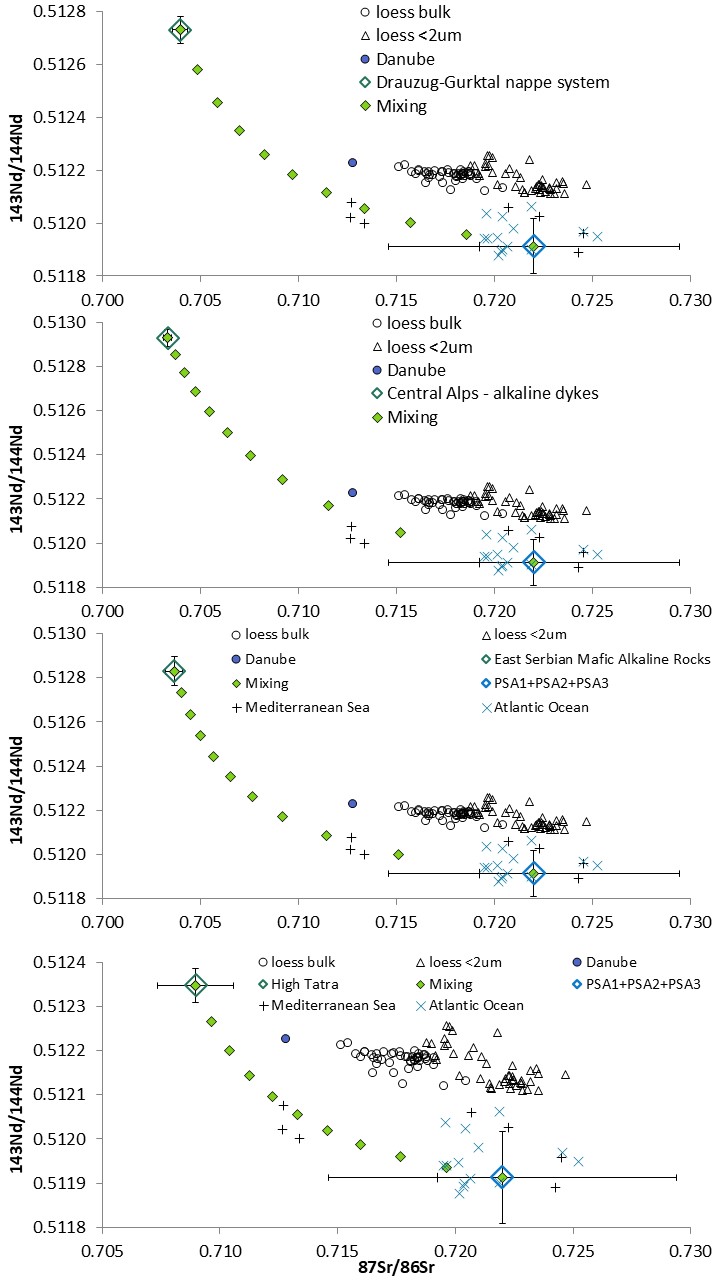

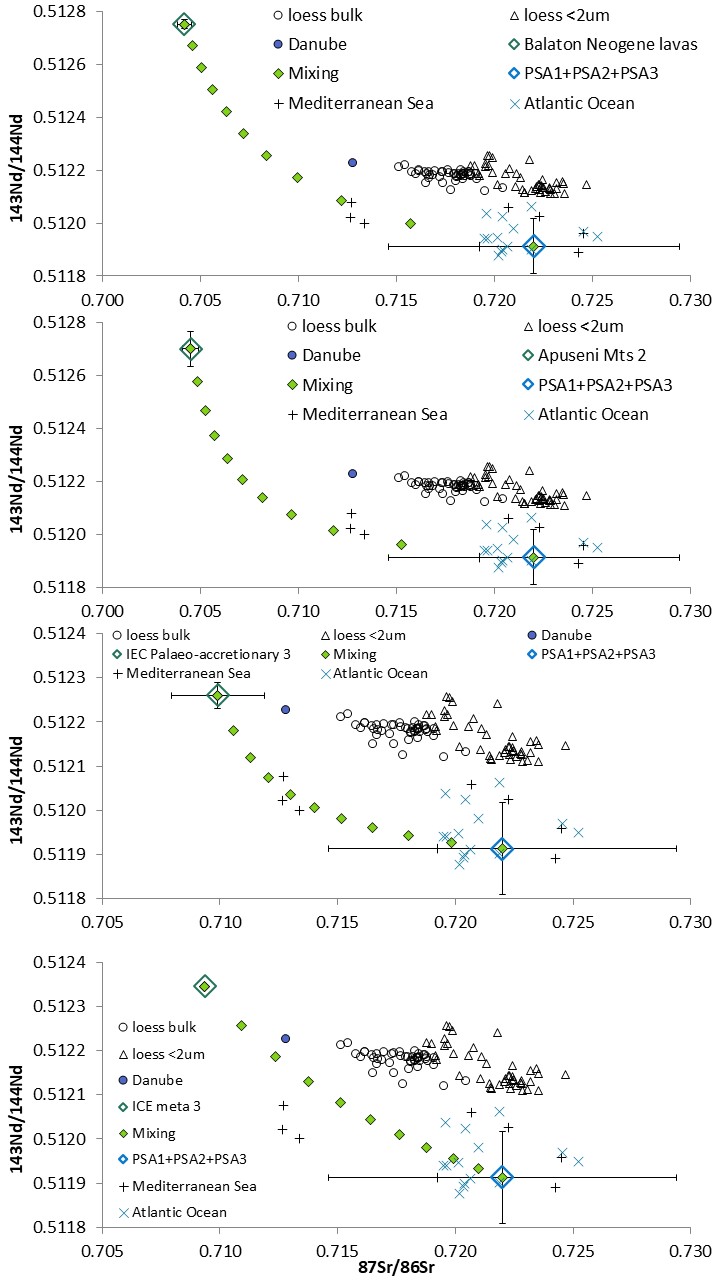

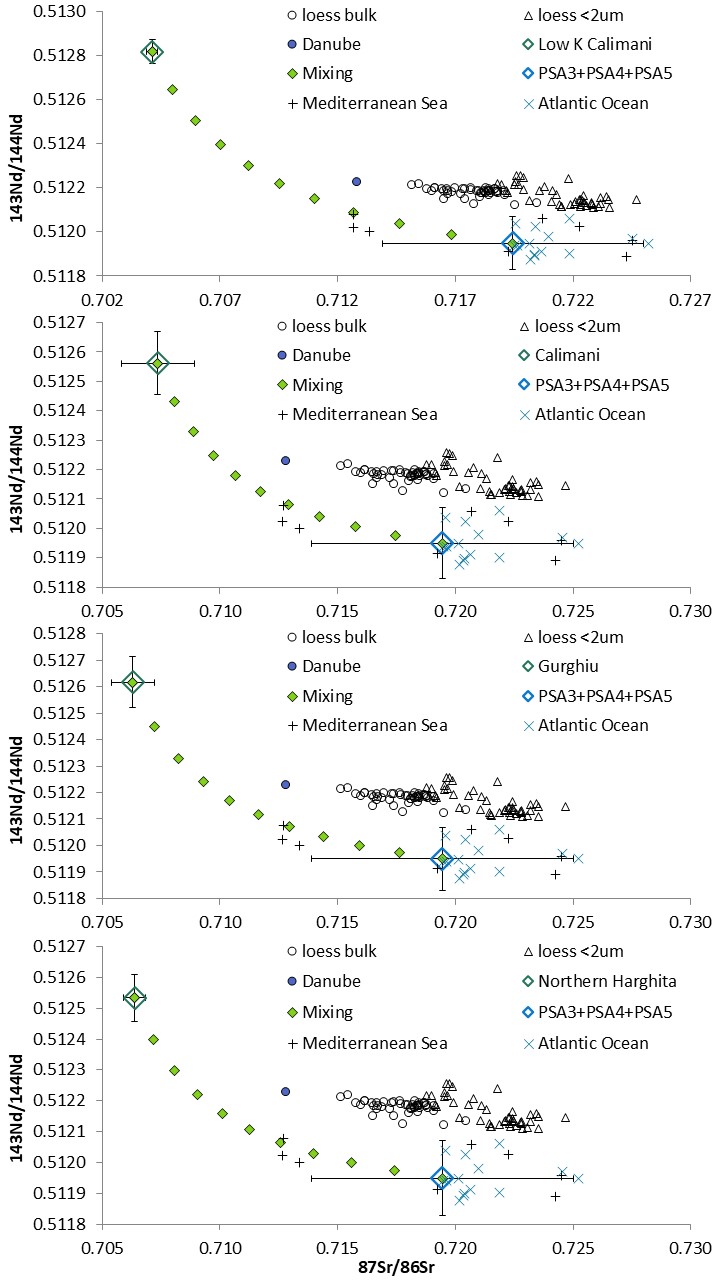

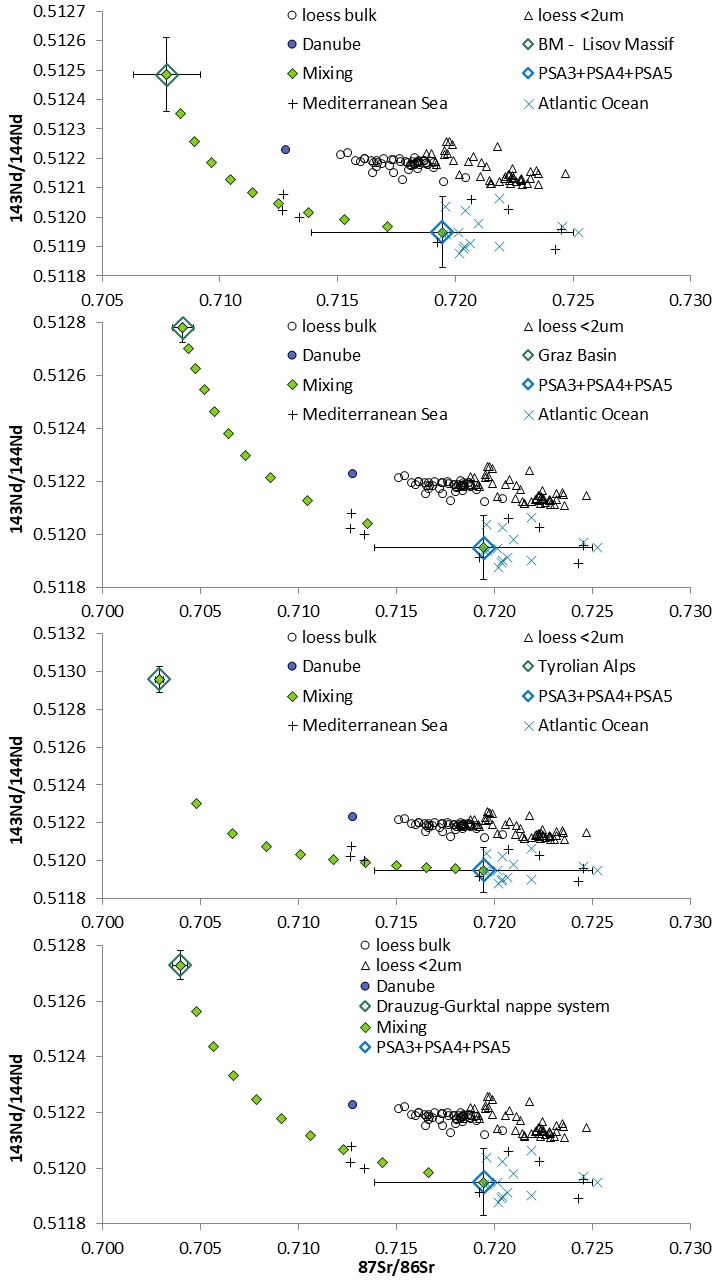

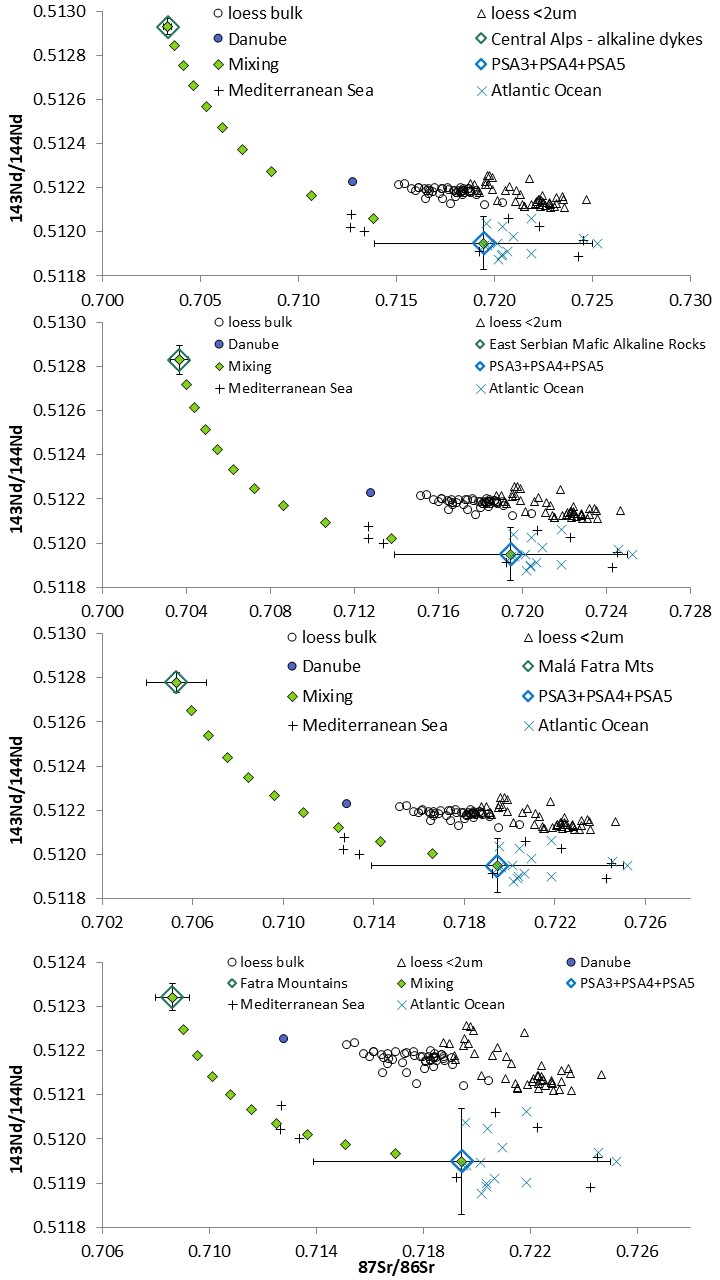

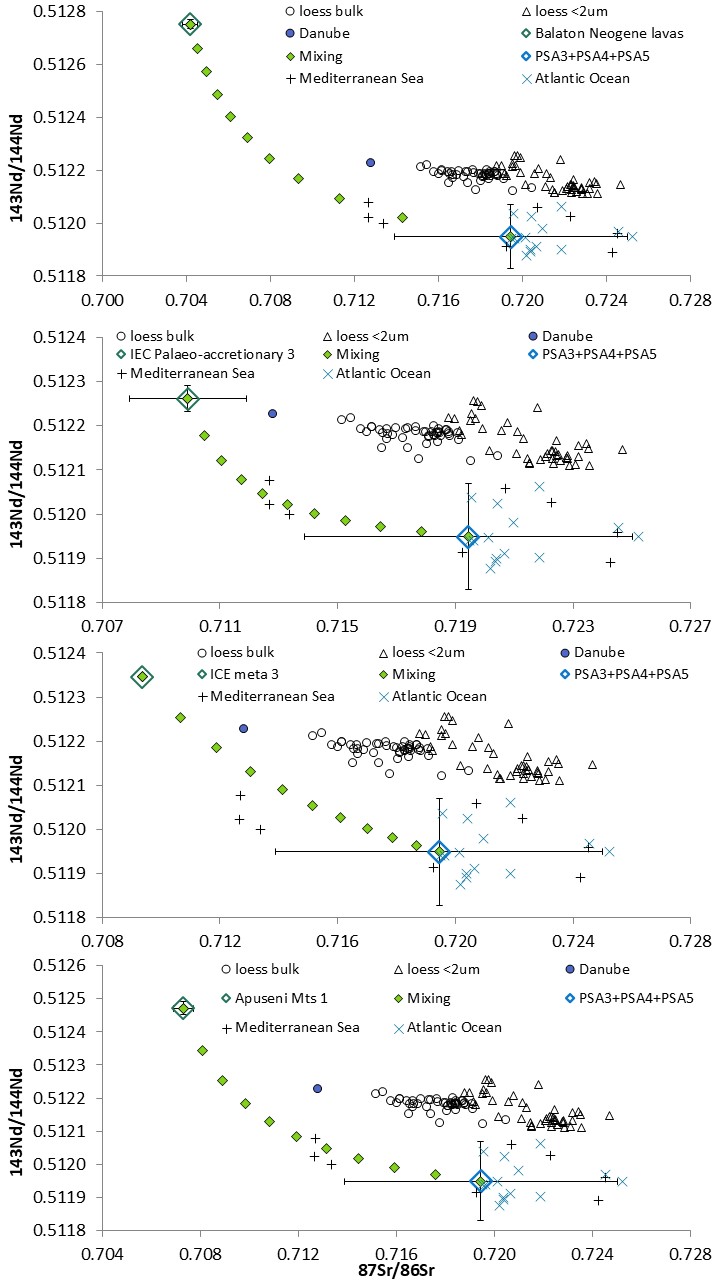
**
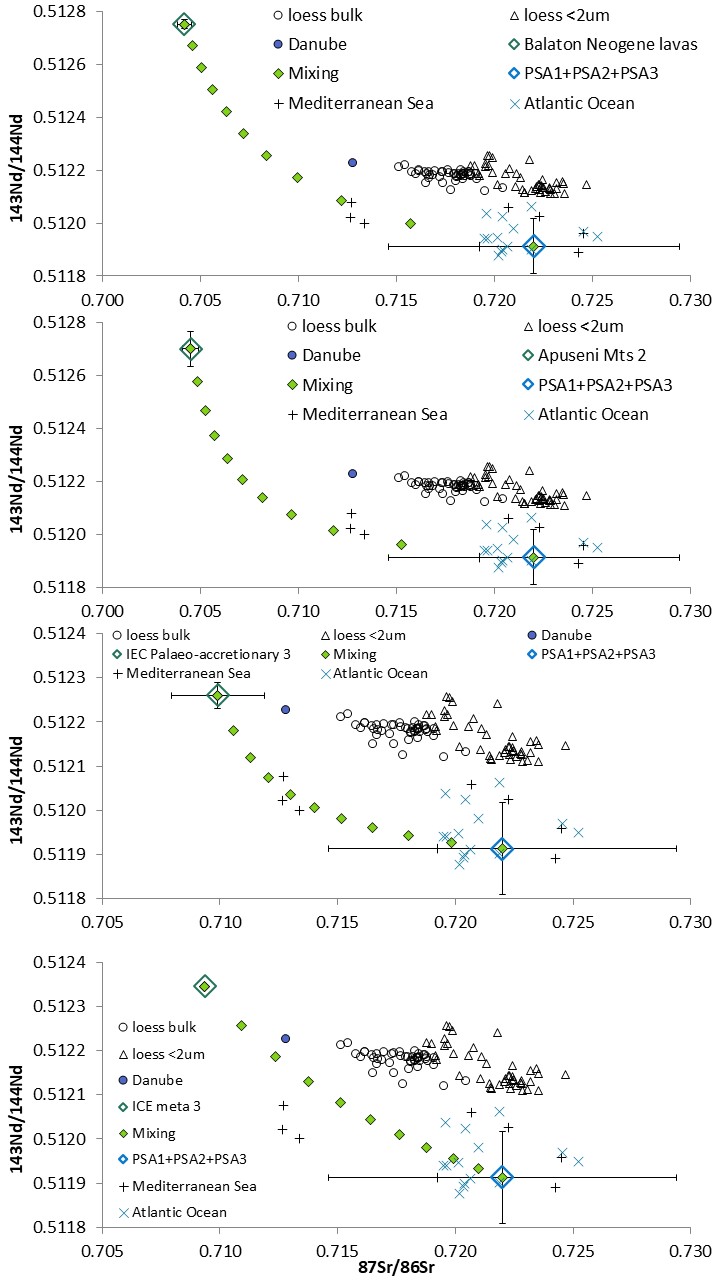

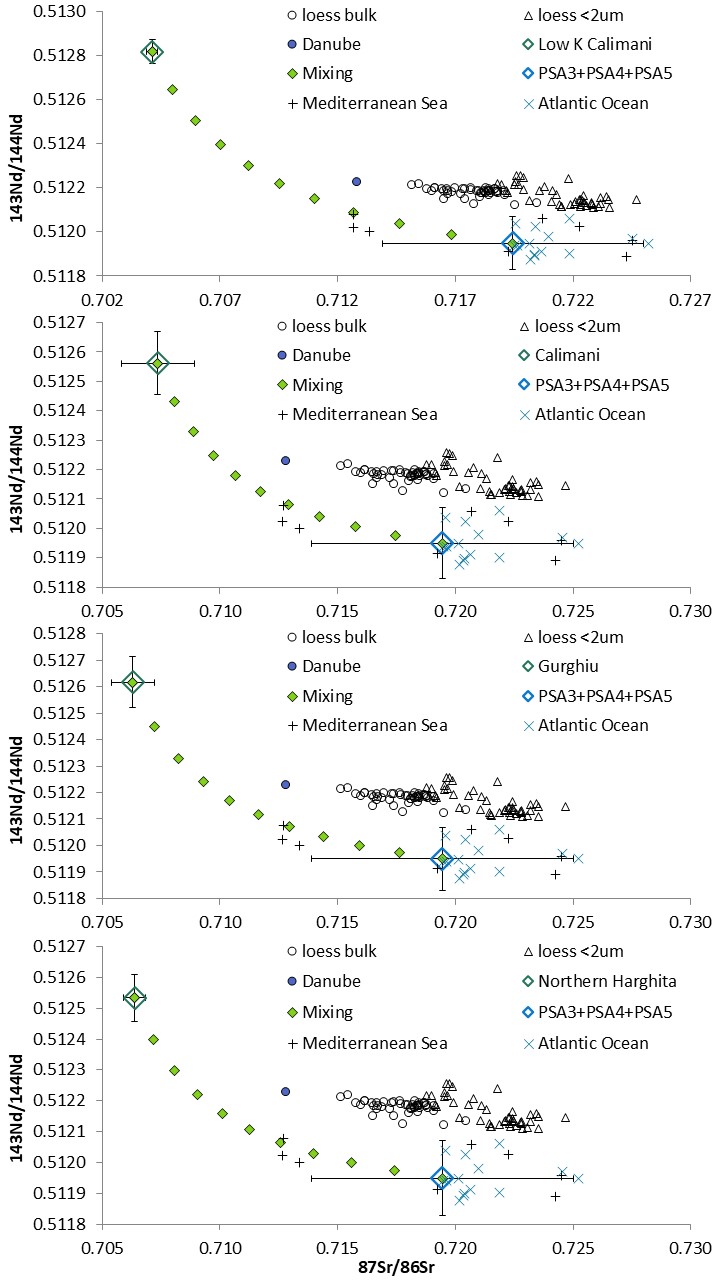

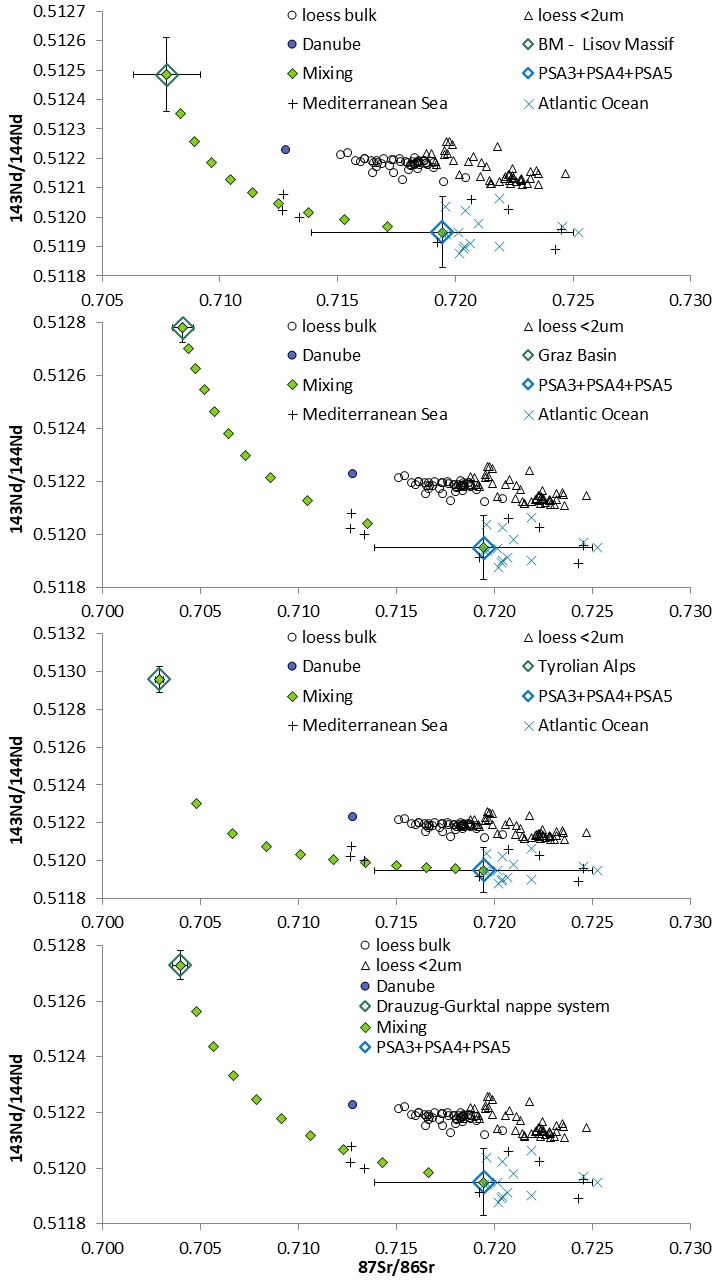

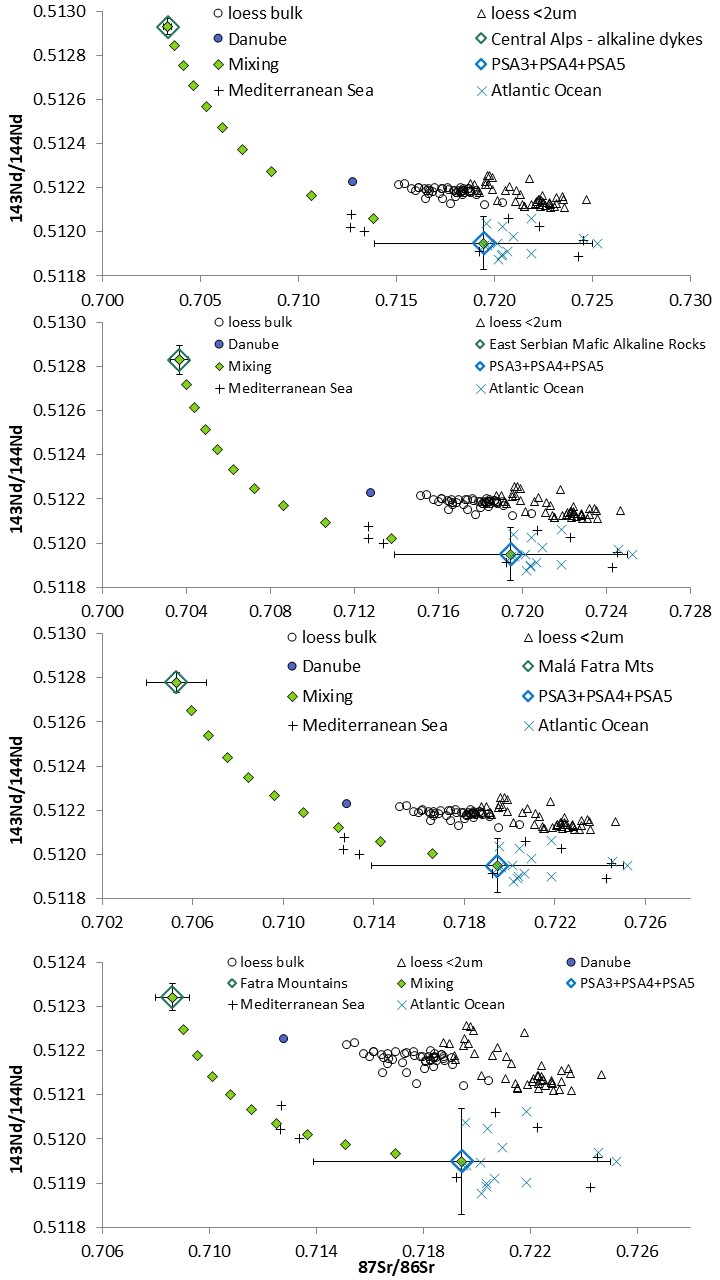

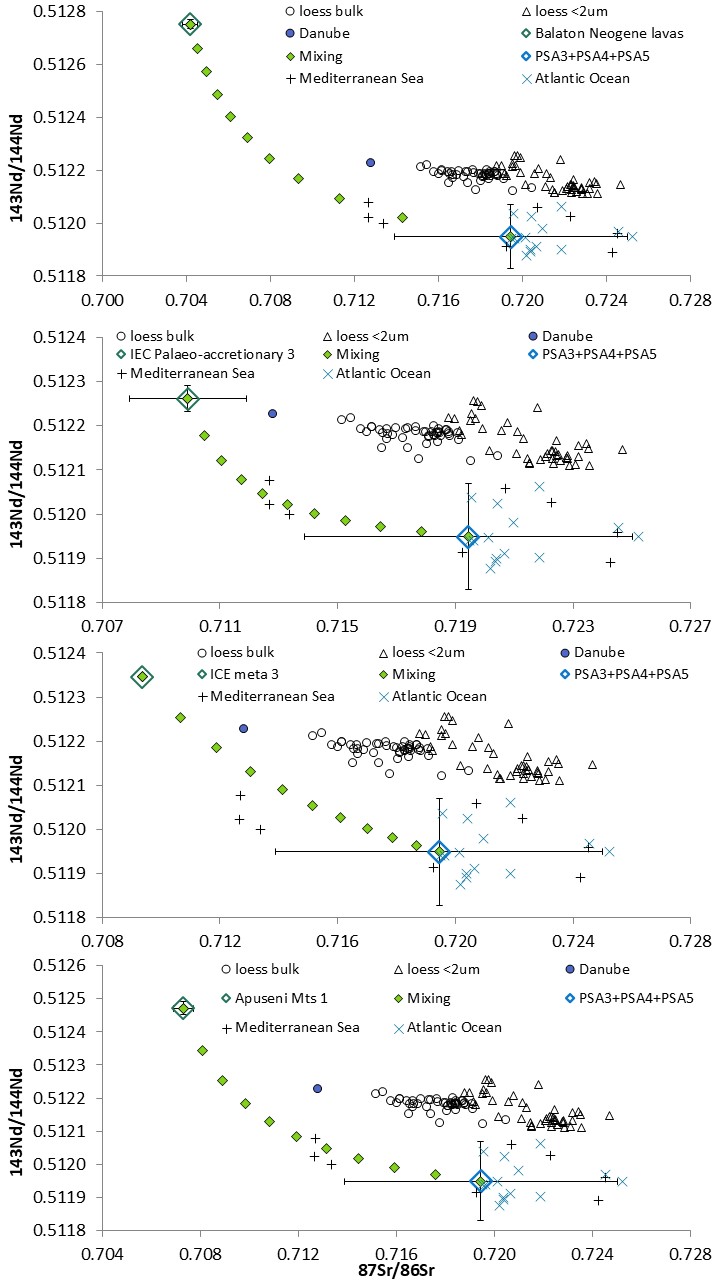

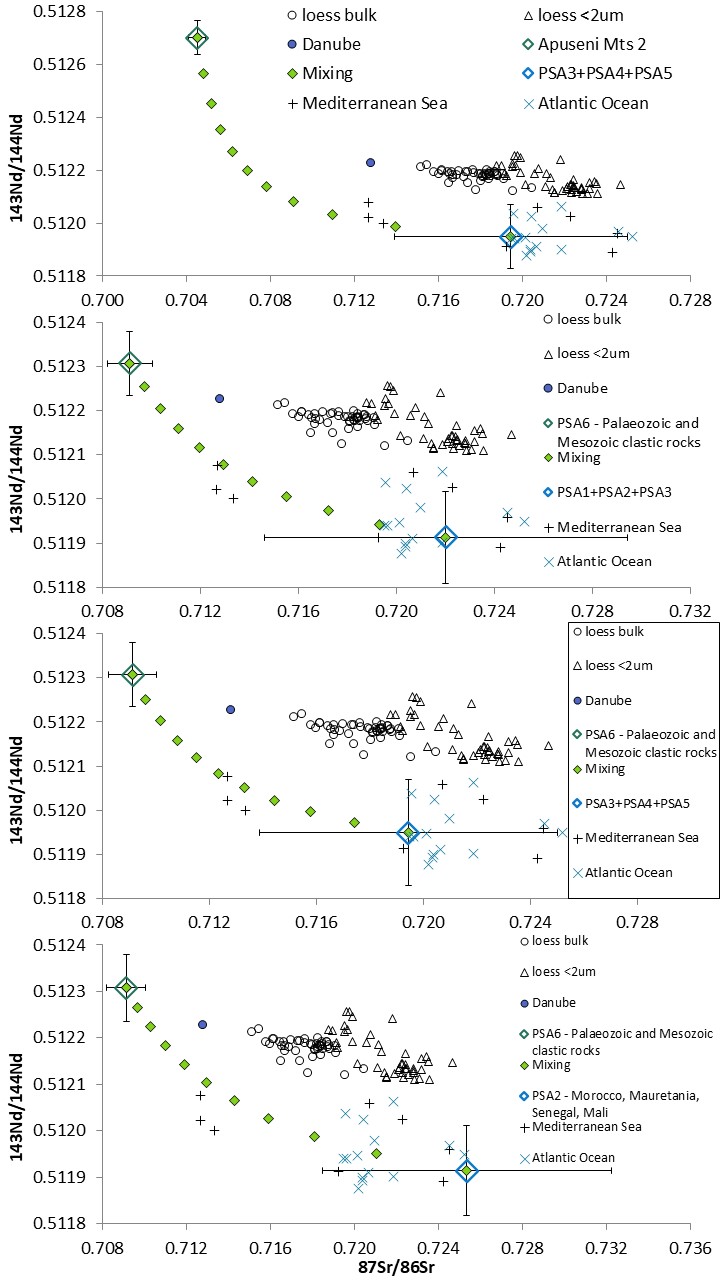
**

**Figure S5.** Mixing lines between averaged values of bedrock and PSAs for a range of explored combinations.

# **Supplementary Figure 6**


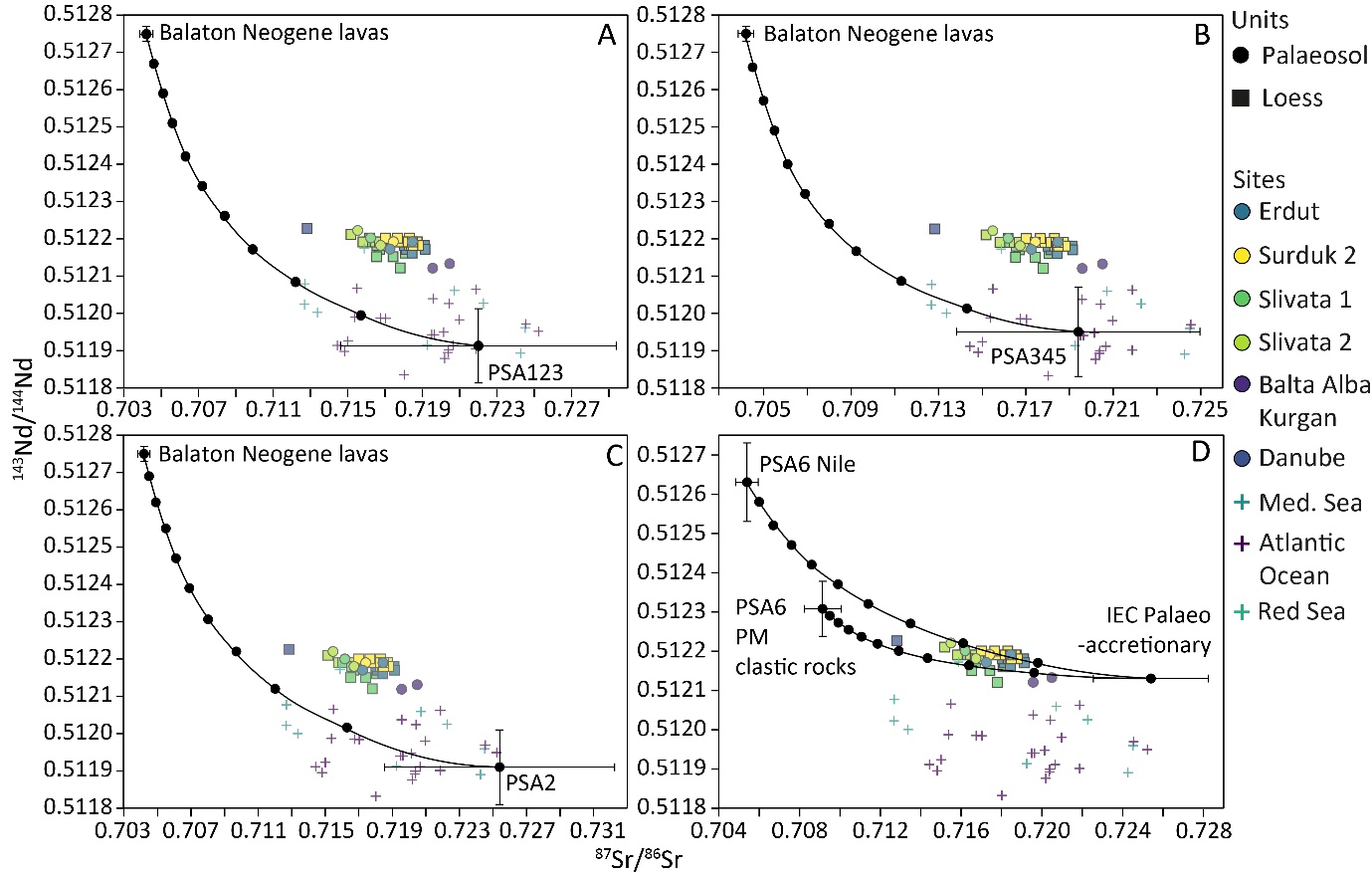


**Figure S6.** Mixing lines between averaged most common dust emitting areas PSA 1, PSA2, PSA 3, PSA4, PSA5 and Neogene volcanic rocks in Balaton lake area (Embey-Isztin et al., 1993) (including the standard deviation of the dataset); D) PSA6 (split into Nile and Palaeozoic and Mesozoic clastic rocks samples) mixed with European flysch. PSA mean points have been calculated only from datasets that have corresponding elemental data (Supplementary Figure 2). In all cases data from dust from Mediterranean Sea and Red Sea aerosols, and Atlantic Ocean surface sediments (Grousset et al., 1998; Revel et al., 2010) are also included. Loess is separated by loess and palaeosol units. Mixing lines calculated following Faure (2001).

# **References**

Abouchami, W., Näthe, K., Kumar, A., Galer, S.J.G., Jochum, K.P., Williams, E.R., Horbe, A.M.C., Rosa, J.W.C., Balsam, W., Adams, D., Mezger, K., Andreae, M.O., 2013. Geochemical and isotopic characterization of the Bodélé depression dust source and implications for transatlantic dust transport to the Amazon basin. Earth Planet. Sci. Lett. 380, 112–123. https://doi.org/10.1016/j.epsl.2013.08.028

Biscaye, P.E., Grousset, F.E., Revel, M., Van Der Gaast, S., Zielinski, G.A., Vaars, A., Kukla, G.J., 1997. Asian provenance of glacial dust (stage 2) in the Greenland Ice Sheet Project 2 Ice Core, Summit, Greenland. J. Geophys. Res. 102, 26765–26781. https://doi.org/10.1029/97JC01249

Embey-Isztin, A., Downes, H., James, D.E., Upton, B.G.J., Dobosi, G., Ingram, G.A., Harmon, R.S., Scharbert, H.G., 1993. The Petrogenesis of Pliocene Alkaline Volcanic Rocks from the Pannonian Basin, Eastern Central Europe. J. Petrol. 34, 317–343.

Faure, G., 2001. Origin of Igneous Rocks. Springer Berlin Heidelberg, Berlin, Heidelberg. https://doi.org/10.1007/978-3-662-04474-2

Gross, A., Palchan, D., Krom, M.D., Angert, A., 2016. Elemental and isotopic composition of surface soils from key Saharan dust sources. Chem. Geol. 442, 54–61. https://doi.org/10.1016/j.chemgeo.2016.09.001

Grousset, F.E., Biscaye, P.E., 2005. Tracing dust sources and transport patterns using Sr, Nd and Pb isotopes. Chem. Geol. 222, 149–167. https://doi.org/10.1016/j.chemgeo.2005.05.006

Grousset, F.E., Parra, M., Bory, A.J.M., Martinez, P., Bertrand, P., Shimmield, G., Ellamn, R.M., 1998. Saharan wind regimes traced by the Sr-Nd isotopic composition of subtropical Atlantic sediments: Last Glacial Maximum vs today. Quat. Sci. Rev. 17, 395–409.

Grousset, F.E., Rognon, P., Coudé-Gaussen, G., Pédemay, P., 1992. Origins of peri-Saharan dust deposits traced by their Nd and Sr isotopic composition. Palaeogeogr. Palaeoclimatol. Palaeoecol. 93, 203–212. https://doi.org/10.1016/0031-0182(92)90097-O

Haliva-Cohen, A., Stein, M., Goldstein, S.L., Sandler, A., Starinsky, A., 2012. Sources and transport routes of fine detritus material to the Late Quaternary Dead Sea basin. Quat. Sci. Rev. 50, 55–70. https://doi.org/10.1016/j.quascirev.2012.06.014

Jewell, A.M., Drake, N.A., Crocker, A.J., Bakker, N.L., Kunkelova, T., Bristow, C.S., Cooper, M.J., Milton, J.A., Breeze, P.S., Wilson, P.A., 2021. Three North African dust source areas and their geochemical fingerprint. Earth Planet. Sci. Lett. 554, 116645. https://doi.org/10.1016/j.epsl.2020.116645

Kumar, A., Abouchami, W., Galer, S.J.G., Garrison, V.H., Williams, E.R., Andreae, M.O., 2014. A radiogenic isotope tracer study of transatlantic dust transport from Africa to the Caribbean. Atmos. Environ. 82, 130–143. https://doi.org/10.1016/j.atmosenv.2013.10.021

Revel, M., Ducassou, E., Grousset, F.E., Bernasconi, S.M., Migeon, S., Revillon, S., Mascle, J., Murat, A., Zaragosi, S., Bosch, D., 2010. 100,000 Years of African monsoon variability recorded in sediments of the Nile margin. Quat. Sci. Rev. 29, 1342–1362. https://doi.org/10.1016/j.quascirev.2010.02.006

Schatz, A.-K., Qi, Y., Siebel, W., Wu, J., Zöller, L., 2015. Tracking potential source areas of Central European loess: examples from Tokaj (HU), Nussloch (D) and Grub (AT). Open Geosci. 7, 678–720. https://doi.org/10.1515/geo-2015-0048

Újvári, G., Klötzli, U.S., Stevens, T., Svensson, A.M., Ludwig, P., Vennemann, T.W., Gier, S., Horschinegg, M., Palcsu, L., Hippler, D., Kovács, J.I., Di Biagio, C., Formenti, P., 2022. Greenland Ice Core Record of Last Glacial Dust Sources and Atmospheric Circulation. J. Geophys. Res. Atmos. 127, 1–23. https://doi.org/10.1029/2022JD036597

Újvári, G., Stevens, T., Svensson, A.M., Klötzli, U.S., Manning, C.J., Németh, T., Kovács, J.I., Sweeney, M.R., Gocke, M., Wiesenberg, G.L.B., Marković, S.B., Zech, M., 2015. Two possible source regions for central Greenland last glacial dust. Geophys. Res. Lett. 42, 10399–10408. https://doi.org/10.1002/2015GL066153

Zhao, W., Balsam, W., Williams, E., Long, X., Ji, J., 2018. Sr–Nd–Hf isotopic fingerprinting of transatlantic dust derived from North Africa. Earth Planet. Sci. Lett. 486, 23–31. https://doi.org/10.1016/j.epsl.2018.01.004
